# Anti-breast cancer synthetic peptides derived from the *Anabastestudineus* skin mucus fractions

**DOI:** 10.1038/s41598-021-02007-6

**Published:** 2021-11-30

**Authors:** Ahmed Abdul Kareem Najm, Ahmad Azfaralariff, Herryawan Ryadi Eziwar Dyari, Babul Airianah Othman, Muhammad Shahid, Nahid Khalili, Douglas Law, Sharifah Sakinah Syed Alwi, Shazrul Fazry

**Affiliations:** 1 Department of Biological Sciences and Biotechnology, Faculty of Science and Technology, Universiti Kebangsaan Malaysia, 43600 Bangi, Selangor Darul Ehsan Malaysia; 2 Department of Food Sciences, Faculty of Science and Technology, Universiti Kebangsaan Malaysia, 43600 Bangi, Selangor Darul Ehsan Malaysia; 3 Department of Earth Sciences and Environmental, Faculty of Science and Technology, Universiti Kebangsaan Malaysia, 43600 Bangi, Selangor Darul Ehsan Malaysia; 4 Innovative Center for Confectionery Technology (MANIS), Faculty of Science and Technology, Universiti Kebangsaan Malaysia, 43600 Bangi, Selangor Darul Ehsan Malaysia; 5 Faculty of Health and Life Sciences, Inti International University, Persiaran Perdana BBN Putra Nilai, 71800 Nilai, Negeri Sembilan Malaysia; 6 Department of Biomedical Science, Faculty of Medicine & Health Sciences, Universiti Putra Malaysia, 43400 UPM Serdang, Selangor Darul Ehsan Malaysia; 7 Chini Lake Research Centre, Faculty of Science and Technology, Universiti Kebangsaan Malaysia, 43600 Bangi, Selangor Darul Ehsan Malaysia

**Keywords:** Cancer, Cell biology, Drug discovery, Molecular biology

## Abstract

Previous study has shown the antimicrobial activities of mucus protein extracted from *Anabas* *testudineus*. In this study, we are interested in characterizing the anticancer activity of the *A.* *testudineus* antimicrobial peptides (AMPs). The mucus was extracted, fractioned, and subjected to antibacterial activity testing to confirm the fish's AMPs production. The cytotoxic activity of each fraction was also identified. Fraction 2 (F2), which shows toxicity against MCF7 and MDA-MB-231 were sent for peptide sequencing to identify the bioactive peptide. The two peptides were then synthetically produced and subjected to cytotoxic assay to prove their efficacy against cancer cell lines. The IC_50_ for AtMP1 against MCF7 and MDA-MB-231 were 8.25 ± 0.14 μg/ml and 9.35 ± 0.25 μg/ml respectively, while for AtMP2 it is 5.89 ± 0.14 μg/ml and 6.97 ± 0.24 μg/ml respectively. AtMP1 and AtMP2 treatment for 48 h induced breast cancer cell cycle arrest and apoptosis by upregulating the p53, which lead to upregulate pro-apoptotic BAX gene and downregulate the anti-apoptotic BCL-2 gene, consequently, trigger the activation of the caspase-3. This interaction was supported by docking analysis (QuickDBD, HPEPDOCK, and ZDOCK) and immunoprecipitation. This study provided new prospects in the development of highly effective and selective cancer therapeutics based on antimicrobial peptides.

## Introduction

Breast cancer poses a crucial public health concern, which needs more molecular-level study to identify its prognosis and clinical care^1,2^. It has become apparent in recent years that breast cancer does not constitute a single disease but rather a variety of molecularly distinct tumours that emerge from the breast's epithelial cells. Cell lines tend to be a key element in the molecular diagnosis of breast cancer, as they can be widely used in many aspects of laboratory research and particularly in cancer research as in-vitro models^3^. A broad range of marine animal's organisms has been identified as a source of possible therapeutic and medicinal qualities^4–6^. Fishes, algae, and sponges were reported to contain anticancer, antiproliferative, antioxidant and antimicrobial effects^7,8^. Besides, a variety of bioactive substances have extracted from fish muscle proteins, peptides, collagen and gelatine, fish oil, and fishbone as potential bioactive compounds^4,9^.

Antimicrobial peptides (AMPs) had garnered the attention of many researchers due to its ability to both inhibit microbes and neoplasm^10–12^. Evidence showed that AMPs besides their antimicrobial activity they demonstrate antitumor roles, possibly in the form of a multifunctional multicellular organism host protection mechanism^13–16^. Hsu and co-workers have extracted two AMP from dull fish muscle by enzymatic hydrolysis with papain and protease against breast cancer human cell line MCF7^17^. Hydrophobic AMPs obtained from anchovy fish sources has been able to cause apoptosis in human U937 lymphoma cells by growing caspase-3 and caspase-8 activity^18^. Tilapia (*Oreochromis* *mossambicus*) hepcidin TH2-3 has been tested on multiple lines of tumour cells, which showed that human fibro sarcoma (HT1080 cell line) proliferation was inhibited^19^. Meanwhile, Chang and co-workers have examined an antimicrobial peptide (TH1-5) to determine antitumor activity in cancer cells, including human cervix adenocarcinoma cells (HeLa), human hepatocellular carcinoma cells (HepG2), human fibro sarcoma cells (HT1080), Cercopithecus aethiops kidney cells (COS-7), and human kidneys cells (WS-1)^20^.

Climbing perch *Anabas* *testudineus* fish are common in Asia, where they are used source of food. The epidermis is characterized by a thick coat of slime containing lipids, proteins, mucopolysaccharides, and other enzymes which are essential in ensuring the preservation of moisture in the skin, thus facilitating the survival of fish at the harsh environment, especially during land movement^21,22^. Evidence showed that proteins in bioactive crude extracted from *A.* *testudineus* have potential antibacterial and haemolytic activities^21^. However, the physicochemical parameters which determine some of the *A.* *testudineus* AMPs activities against cancer cells are still unknown, considering that the characteristics of AMPs and anticancer peptide (ACPs) are very similar^23,24^. Due to these facts, developing research in this field is important in addressing such issues^1^. Thus, this study aimed to examine the antibacterial ad anticancer activities for mucus fraction extracts of *A.* *testudineus* fish. More importantly, we investigate the role and pathway of these fractions in inhibiting the proliferative of breast cancer cell lines. We also investigate the change in gene expression resulted from the mechanism of action of these fractions.

## Materials and methods

### Sample preparation

A total of 30 climbing perch with an average weight of 300 g was obtained from a local fish farm in Jelebu, Negeri Sembilan, Malaysia. The fishes were acclimatised for 7 days (in a group of three) in a 26 × 17 × 19 cm plastic aquarium at a temperature of 28 °C, at pH range between 6 and 6.5. The water used in this experiment was dechlorinated tap water. The fish were fed a commercial pelleted diet (ad libitum composed of protein, 18–50%; lipids, 10–25%; carbohydrate, 15–20%; ash, < 8.5%; phosphorus, and trace amounts of vitamins, minerals, and water) for the entire period of the experiment. All experiments conducted were approved by Universiti Kebangsaan Malaysia (UKM) animal ethics committee (Ethics code: FST/2019/MOHD SHAZRUL FAZRY/25 SEPT./1034-SEPT.-2019-FEB-2020). All the methods accordance with ARRIVE guidelines.

### Crude mucus collection

The Mucus of the selected *A.* *testudineus* was extracted as described by Ross et al.^25^ with slight modifications. The selected fish were anesthetised using Tricaine methane-sulfonate (MS-222). The epidermal mucus (EM) was collected from the dorsal side using a cell scraper, then transferred to a 15 ml falcon tube containing 3 ml buffer solution (pH 7.4) having 0.013 M Tris, 0.12 M NaCl, and 0.003 M KCl. The collected mucus was centrifuged at 1000×*g* for 10 min at 4 °C and stored at − 80 °C for further use.

### Mucus purification

The frozen EM was thawed and centrifuged at 1000×*g* for 10 min at 4 °C to remove insoluble particles. The supernatant was collected, and once again centrifuged at 2000×*g* for 10 min at 4 °C. The supernatant was collected and freeze dried (Labconco 74200-30). The lyophilised EM powder were weighted and kept in − 80 °C for further use^25^. To prepare purified EM stock solution, 0.5 g of EM powder was resuspended in 5 ml of distilled water^25^, centrifuged at 2500×*g* for 5 min at 4 °C, and used for further studies.

### Mucus protein content

The protein content of EM was quantified using Bradford's (1976) technique. The purified EM stock solution (0.5 ml) was mixed with 1.5 ml of Bradford reagent. The samples were incubated at room temperature for 10 min and then the absorbance was read at 595 nm by using Shimadzu 160 UV–Vis double beam Spectrophotometer. The bovine serum albumin (BSA) was used as the standard.

### Fractioning of crude mucus of *A. testudineus*

The crude mucus was fractionized as described by Yang et al.^26^ with slight modifications. The 0.5 g purified freeze-dried mucus was resuspended in 5 ml distilled water and loaded on to Sephadex G-25 column (10 cm, 50 ml bed volume), then equilibrated with 50 ml of 0.1 M acetate buffer, pH 4.3 in the cold room using a Dynamix peristaltic pump to control the flow rate. After equilibrating 5 ml of sample is loaded onto the column, which is then washed with tris-base buffer at pH 8 to elute the sample skin mucus. Each fraction (0.5 ml) was collected in microcentrifuge tubes after concentrated by ultrafiltration using viva spin nominal molecular-mass limit of 6000 kDa. The protein concentration of the extract is determined by Bradford assay as described in “Mucus protein content”. Dextran blue was used as a positive control^26^. The fraction protein profile was detected by sodium dodecyl sulphate–polyacrylamide gel. The protein profile was run in 12% sodium dodecyl sulphate–polyacrylamide gel electrophoresis (SDS-PAGE). A mixture of 4.0 ml of 30% acrylamide, 2.6 ml of 1.5 M Tris buffer (pH 8.8), 3.2 ml of water, 100 l of 10% SDS, 100 μl of 10% APS, and 10 μl of TEMED was mixed to make the 12% running gel. Then, a mixture of 2.6 ml of 0.5 M Tris buffer (pH 6.8), 5.86 ml of water, 1.34 ml of 30% acrylamide, 100 μl of 10% SDS, 100 μl of 10% APS, and 10 μl of TEMED were applied to prepare a 4% stacking gel. The protein was solubilized at 1 × volume of reducing sample buffer that was composed of 1 M Tris–HCl (pH 6.8), 50% glycerol, 10% SDS, 5% β-mercaptoethanol, water, and 1% bromophenol blue. The buffer was incubated for 5 min at 95 °C and kept on ice before being loaded to the gel. The gel was applied to a Bio-Rad electrophoresis apparatus for 2 h at 30 V throughout preliminary voltage, followed by 150 V until 2 mm from the gel base. The obtained bands were observed for the Coomassie Brilliant Blue G-250 stains.

### Identification of peptide sequences

Each protein sub-fractions from “Fractioning of crude mucus of *A. testudineus*” were hydrolysed by trypsin at an enzyme to the protein ratio of 1:50 at 37 °C for 24 h, except the lower than 3 kDa sub-fraction. The peptides were dried by vacuum centrifuge and kept at − 80 °C before conducting mass spectrometric analysis. The peptide sequence was identified by using LC–MS. In general, acetonitrile was used as the organic modifier in all separations, with concentrations ranging between 40 and 80%, by using Karpievitch and co-workers’ method^27^. The aqueous portion of the mobile phase consisted of water, ammonium acetate buffer. Flow rates were 0.4 to 0.6 ml/min for LC/MS detector and 1.0 ml/min for UV detector. All fractions were dissolved in a 50/50 mixture of water and methanol. Stock solutions, 1 mg/ml of dissolved fraction was stored in a freezer. Working solutions were made to contain 10 µg/ml of each fraction of interest. The C_18_ separations of the fractions were conducted with the MS compatible mobile phases (aqueous acetic acid or acetate buffers with acetonitrile as the buffer) and similar flow rate conditions (0.5 ml/min vs 0.6 ml/min). UV detection was at 214 nm. This assay has been done in Liquid Chromatography-Mass Spectrometry (LCMS) Platform, LCMS Laboratory, Jeffrey Cheah School of Medicine and Health Science, Monash University Malaysia.

### Bioinformatics prediction of peptides

**Figure 1:**
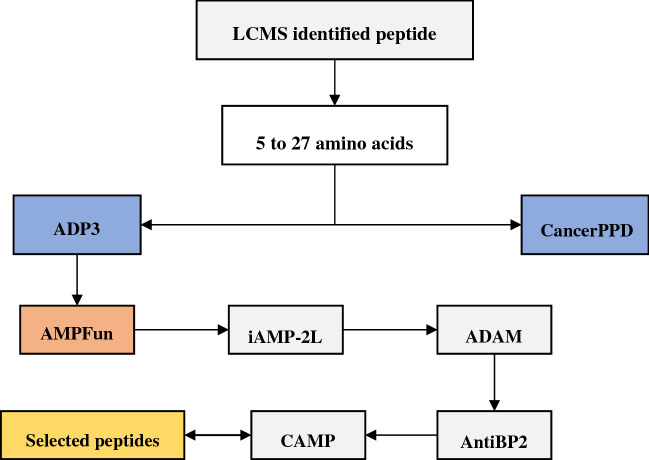
Workflow for bioinformatics prediction of antimicrobial peptides.

The selected potential peptides after fractionization were then blasted against the antimicrobial peptides database: AMPfun (http://fdblab.csie.ncu.edu.tw/AMPfun/index.html)^28^; iAMP-2L (http://www.jci-bioinfo.cn/iAMP-2L)^29^; and CAMP-R3 (http://www.camp3.bicnirrh.res.in/)^30^. These peptides were screened for putative antimicrobial peptides by using consensus prediction. Each peptide was submitted for the prediction of two programs; ADAM (http://bioinformatics.cs.ntou.edu.tw/ADAM/)^31^ and ADp3 (http://aps.unmc.edu/AP/)^32^ for antimicrobial peptide prediction based on amino acid composition, conserved features, and physicochemical properties. Peptides positively predicted by both programs were considered putative antimicrobial peptides with anticancer and antimicrobial activity. These peptides were checked for toxicity and antimicrobial activity (Fig. 1).

### Peptide synthesis

Two potential peptides were selected to be synthesized in-vivo. After select based on the amino acids length and predict peptide functions through in-silico database as described in “Bioinformatics prediction of peptides”. We select two peptides based on molecular weight, the number of amino acids, and the net charge of these peptides as described in Table 1. These two peptides were synthesized and provided by 1st BASE Co., Ltd. (Singapore).

**Table 1:** Details on bioinformatics prediction of antimicrobial peptides from databases.

Peptide sequences	ADP3			AMPFun			iAMP-2L	ADAM	Cancer PPD	CAMP R3	AntiBP2		
	Length	Hydrophobic (%)	Net charge	Anti-cancer	Gram+	Gram−	Type	Type	No data	Type	N-terminus	C-terminus	NC-terminal
GNLNKEMSSAPIVGQPSIPGGPVR	24	29	1	No 0.0042	No 0.5167	No 0.4924	NON-AMPs	NON-AMPs		NAMP	Yes	Yes	Yes
GALALAPTGEVYDIEIDTL	19	47	− 4	No 0.0018	No 0.3667	No 0.2242	NON-AMPs	NON-AMPs		AMP	Yes	No	No
AGVASVESSSGEAF	14	42	− 2	No 0.0478	No 0.4833	No 0.4778	AMPs	AMPs		NAMP	NA	NA	NA
THPPTTTTTTTTTTTTTAAPATTT	24	12	0.25	Yes 0.2816	Yes 0.5667	Yes 0.6051	AMPs	AMPs		NAMP	Yes	No	No
ACGSSTRSYAMIIDA	15	46	0	No 0.155	No 0.35	No 0.5075	AMPs	AMPs		NAMP	No	No	Yes
TNSYPVFGAGGGEYETI	17	23	− 2	No 0.05	No 0.391	No 0.380	AMPs	AMPs		AMP	No	Yes	No
TGIATSGLATFTLHTGSLAPAT	22	40	0.25	Yes 0.4292	Yes 0.6333	Yes 0.7233	AMPs	AMPs		NAMP	No	No	Yes

### Disc diffusion method

The antimicrobial activity of protein fractions and synthetic peptides obtained from *A.* *testudineus* fish was tested against human pathogens (*Escherichia* *coli*, *Pseudomonas* *aeruginosa*, *Bacillus* *subtilis*, and *Bacillus* *cereus*). The selected microbial was spread on nutrient agar plates by using cotton bud and incubated for 24 h at 37 °C^33^. The functioned discs were prepared by adding 20 μl (1000 µg/ml) of each sample (fractions and peptides) diluted in 10% ethanol on a 6 mm blank antibiotic disc. Then, the disc was placed onto the nutrient agar plate of the bacterial culture and incubated at 37 °C for 24 h. A standard antibiotics disk (10 µg/ml streptomycin) was used as the positive control while a blank disc as negative controls. After the incubation, the inhibition zones that appeared around the discs were measured to the nearest millimetre (mm).

### Cytotoxicity effect

#### Cell lines

Human breast cancer cell line (MCF7), human adenocarcinoma cell line (MDA-MB-231) and human skin new-born for skin fibroblast (HS27) were used in this study to determine the cytotoxicity. Cancer and normal cell lines used in this study were purchased from the American Type Culture Collection Organization (ATCC). Dulbecco's Modified Eagle Medium (DMEM) supplemented with 10% FBS and 1% penicillin. The cells were incubated at 37 °C in CO_2_ 5% and humidity 85 to 95%.

#### Cell cytotoxicity assay

Cell cytotoxicity was performed using 3-(4, 5-dimethylthiazol-2-yl)-2, 5-diphenyltetrazolium bromide (MTT) assay^34^. As the confluence of the cells in the flask cell reached 80%, the medium was discarded, and the cells were washed with 1× phosphate buffer saline (PBS) three times. The trypsin was added for 5–7 min, to unleash cells from the flask wall, then 5 ml of the medium was added to the flask, mixed well, transported into a new tube, and centrifuged for 5 min at 2500×*g*, then the supernatant was discarded. A haemocytometer chamber was used to count the cell. A 100 μl of the cell suspension with concentration of 1 × 10^5^ were seeded into 96 well plates^35^.

After 1 day of incubation, 100 μl of extract was added into each well of 96 well plates at concentrations 1–10 μg/mL and incubated for 24 h and 48 h respectively. The cell cytotoxicity was measured by adding 10 μl of MTT reagent into the cell suspension and left for 4 h. Finally, the content of the wells was discarded, and 200 μl DMSO (100%) was added to each well and left for 15 min. The graphs are representing cell viability (%) by measuring the absorbance of optical density (590 nm) with a microplate reader.

### Cell cycle analysis

The cells were seeded into 6 well plates at 1 × 10^6^ cells/well and incubated for 24 h. After incubation, the cells were treated with the fraction for 24 h, fixed in 70% ethanol, and stained with FxCycle Propidium Iodide (PI)/RNase Staining Solution (Thermo Fisher #F10797). Flowcytometry BD-FACSCanto II (BD Bioscience, Singapore), was used to read the results. The cell cycle histogram was analysed with modified LT V.4^36^. Three biological replicates have been used. The difference in cell cycle checkpoints was then computed in comparison to the cells without treatment.

### Determination of cell apoptosis

#### DNA fragmentation assay

Lee and co-worker’s method^37^ was used to detect DNA fragmentation. The cell cultures were collected from the flask, transferred to a 15 ml tube, and centrifuged at 2800×*g* for 5 min. The supernatant was discarded, then 500 μl lysis buffer (pH 7.4), contain 1 ml 0.5 M Tris–HCl 3 ml 0.5 M NaCl, 0.4 ml 0.05 M EDTA, 0.03 ml Triton X-100, 0.03 ml NP40, 0.5 ml 0.05 M, 0.5 ml Sodium fluoride, 0.02 ml 0.5 M β-glycerophosphate, 0.02 ml 0.05 M Sodium orthovanadate, and 0.05 M PMSF was added, and incubated at room temperature for 10 min. The lysate cells were harvested and transferred into new tubes, then incubated at 65 °C for 5 min. After cooling at room temperature for 5 min, 700 μl chloroform-isoamyl alcohol was added, and then centrifuged at 16,000×*g* for 5 min. The supernatant was discarded, and the pellet was air-dried for 30 min. Then, the dried DNA was dissolved in 50 μl distilled water. A spectrophotometer Nanodrop was used to quantify the extracted DNA concentration. The DNA samples were electrophoresed on a 1.5% agarose gel containing 1 μl/100 ml SYBR-Safe DNA gel stain (Invitrogen, catalogue number: S33102).

#### Annexin V-FITC apoptosis

This experiment used a kit (Elabscience E-CK-A211). The cells (1 × 10^6^ cells) were seeded in a T25 culture flask and one prepared as control (unstained, Annexin and propidium iodide only). After 48 h of incubation, the cells were trypsinized and collected by centrifugation at 1000×*g* for 5 min. The collected cells were washed twice with PBS and centrifuged (1000×g, 5 min, at room temperature). Each pellet (~ 1 × 10^6^ cells) was resuspended in PBS (400 µl). The cells were stained using (400 µl of cells + 100 µl of incubation buffer with 2 µl of Annexin [1 mg/ml] and 2 µl of propidium iodide [1 mg/ml])^38^. Flowcytometry BD-FACSCanto II (BD Bioscience, Singapore), was used to read the results. The cell cycle histogram was analysed with modified LT V.4^36^. The experiments were repeated in triplicate.

### RNA isolation and cDNA synthesis

Total RNA was isolated and purified from the MCF7 and MDA-MB-231 cells using the All-Prep DNA/RNA Mini Kit (QIAGENE) following the manufacturer's instructions. The cells lysate was collected and pipetted directly into a QIAshredder spin column placed in a 2 ml collection tube and centrifuged for 2 min at 16,000×*g*. The homogenized lysate was transferred to an All-Prep DNA spin column placed in a 2 ml collection tube, and centrifuge for 30 s at 8000×*g*. All-Prep DNA spin column was transferred into a new 2 ml collection tube and stored at 2 °C for later DNA purification.

For RNA elution, the cell lysate transferred to RNeasy Mine lute spin column followed by added 350 µl of 70% ethanol and mixed well by pipetting. Then, 14 µl RNase-free water was added and centrifuged for 1 min at 16,000×*g* to elute the RNA. The concentration and purity of the RNA were quantified using spectrophotometer (Nanodrop 1000; Thermo Fisher Scientific, Wilmington, DE, USA). The 0.5 μg of total RNA was used as a template using the RT^2^ First Strand kit (Qiagen, Valencia, CA, USA), following the manufacturer's instructions. A spectrophotometer Nanodrop was used to quantify the extracted RNA concentration.

#### RT^2^ Profiler™ PCR array

Pathway-focused gene expression profiling was performed using a 96‑well human apoptosis PCR array (RT^2^ Profiler PCR array—PAHS-apoptosis, Human PCR array, Qiagen, USA). In this array, each well contains all the components required and designed to generate single, gene-specific amplicons, testing the expression of 84 genes related to breast cancer pathways (apoptosis, metabolism, cell cycle, and DNA repair), plus 5 housekeeping genes. Each RT^2^ Profiler PCR array plate also includes controls for data normalization, genomic DNA contamination detection, RNA sample quality and general PCR performance. The results analysis by using Gene Globe Data Analysis. Genes showed less than 1.5-fold change were neglected.

### Protein–protein interaction (bioinformatics docking)

Protein–protein interactions are essential to immune and cellular activities; however, there is a lack of a physiochemically-determined structure of the compounds^39,40^. These interactions should be enrolled for a better understanding of their molecular basis. To predict the protein-peptide complexes, this study utilized various databases, including QuickDBD (https://www.quickdatabasediagrams.com)^40^, RCSB PDB (https://www.rcsb.org)^41^, HPEPDOCK (http://huanglab.phys.hust.edu.cn/hpepdock)^42^, ZDOCK (https://zdock.umassmed.edu)^39^, and the ligand and protein files were prepared through BIOVIA Discovery Studio Visualizer (DSV) version 20.1.0 software^43^.

**Figure 2:**
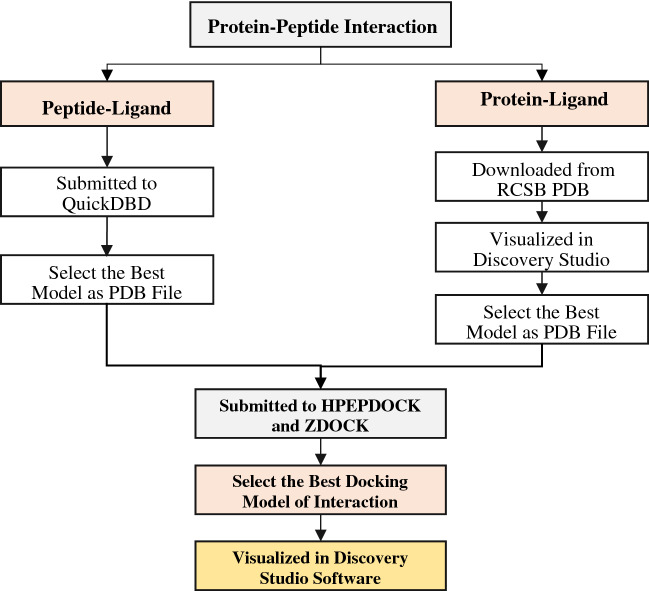
Workflow for protein-peptide docking interaction.

First of all, the peptides’ sequence was submitted to QuickDBD to obtain the best model of the peptides' structure and select the best score model in a PDB file format. Then, the interacted protein in the RT^2^ profiler PCR array was submitted to RCSB PDB to get the PDB file format of the protein structure and its chains. After that, the PDB files of peptides and the protein model were submitted to HPEPDOCK and ZDOCK to obtain and select the best docking model of the protein-peptide complexes based on the Z scoring. Finally, the selected protein-peptide complex model was inserted into BIOVIA DSV software to visualize the structure and obtain the protein-peptide interaction figure with different perspectives (Fig. 2).

### Immunoprecipitation and pull-down assay

#### Total protein extraction

In this stage, the study used Radioimmunoprecipitation assay buffer (RIPA buffer) to lysis and extraction proteins (catalogue number: 89900) for cells’ total protein extraction adapted from Zhu et al.^44^. The culture media was discarded from the flask, and cells were washed twice by using 1× PBS. Subsequently, 1 ml of lysis RIPA buffer was added for 1 × 10^7^ of cells; then, the cells were trypsinized and accumulated by using a cell scraper and kept in 1.5 ml of microcentrifuge tube. The collected cells were incubated at 4 °C for 20 min; after that, the cells were centrifuged at 13,000×*g* for 20 min at 4 °C. Finally, the supernatant consist of the soluble protein was carefully transported to a new microtube, and the cell pellet was discarded. Then the protein kept in − 80 °C for further study. A spectrophotometer Nanodrop was used to quantify the extracted protein concentration.

#### Peptide labelling

For peptide labelling, this study used Biotin Labelling Kit NH_2_ (catalogue number: KA0003) by to adhering the manufacturer's guidelines instructions. 100 μl washing buffer and 200 μg selected AMPs were added in a filtration tube; then, the mixture was pipetted up and down and centrifuged at 8000×*g* for 10 min. Subsequently, DMSO 10 μl was added to NH_2_ reactive biotin and dissolved with vortex. A reaction buffer of 100 μl with 8 μl of NH_2_ reactive biotin was added to a filtration tube, mixed gently, and incubated for 10 min at 37 °C. This was followed by the addition of 100 μl of WS buffer to the tube and centrifugation at 8000×*g* for 10 min and discarding of the filtrate (this step was repeated twice). A 200 μl WS buffer was added to the tube and pipetted 10 times to recover the conjugate. The solution was transferred to a microtube and stored at 5 °C.

#### Immunoprecipitation

The extracted proteins from the cell protein extraction method (see “Total protein extraction”) were pooled with the conjugated peptides from the previous stage (see “Peptide labelling”) to link the cell protein with the peptides using Cheah and Yamada’s technique^45^. In this method, 200 µl of cell proteins were incubated with 20 µl of Streptavidin and kept on a rotator overnight at 4 °C to isolate biotinylated proteins from cell lysates. The beads were firstly washed with lysis buffer and transferred into a new tube and washed again with 2% SDS in 50 mM Tris HCl pH 7.4. Secondly, the biotinylated proteins were eluted from these beads by using 30 µl of 25 mM biotin for 5 min at 95 °C. After considerable wash, binding proteins were eluted by boiling in SDS sample buffer composed of 1 M Tris–HCl (pH 6.8), 50% glycerol, 10% SDS, 5% β-mercaptoethanol, and water. By using 50 mM DTT, the bait peptides with their bound proteins were removed from the beads. Thirdly, a sample buffer 4×, 80 µl was added to the beads with 200 mM Tris HCl pH 6.8, 40% glycerol, 8% SDS, 8% β-mercaptoethanol, and 0.04% bromophenol blue, and heated at 95 °C for 5 min. Subsequently, the biotinylated proteins were detected by sodium dodecyl sulphate–polyacrylamide gel. The protein profile was run in 12% sodium dodecyl sulphate–polyacrylamide gel electrophoresis (SDS-PAGE). A mixture of 4.0 ml of 30% acrylamide, 2.6 ml of 1.5 M Tris buffer (pH 8.8), 3.2 ml of water, 100 l of 10% SDS, 100 μl of 10% APS, and 10 μl of TEMED was mixed to make the 12% running gel. Then, a mixture of 2.6 ml of 0.5 M Tris buffer (pH 6.8), 5.86 ml of water, 1.34 ml of 30% acrylamide, 100 μl of 10% SDS, 100 μl of 10% APS, and 10 μl of TEMED were applied to prepare a 4% stacking gel. The protein was solubilized at 1 × volume of reducing sample buffer that was composed of 1 M Tris–HCl (pH 6.8), 50% glycerol, 10% SDS, 5% β-mercaptoethanol, water, and 1% bromophenol blue. The buffer was incubated for 5 min at 95 °C and kept on ice before being loaded to the gel. The gel was applied to a Bio-Rad electrophoresis apparatus for 2 h at 30 V throughout preliminary voltage, followed by 150 V until 2 mm from the gel base. The obtained bands were observed for the Coomassie Brilliant Blue G-250 stains. were run on the SDS page to check the protein profile and then run with LC–MS–MS to identify the protein sequence.

### Statistical analysis

All data were expressed as the mean ± standard deviation (SD) of the values obtained from at least three replicates. A Statistical Package for the Social Sciences Program (SPSS) version 23 was used to conduct one-way analysis of variance (ANOVA), where the p-value < 0.05 was considered to be statistically significant.

### Ethical approval

All authors declared that all methods and protocols were carried out in accordance with relevant guidelines and regulations. All experiments conducted were approved by Universiti Kebangsaan Malaysia (UKM) animal ethics committee (Ethics code: FST/2019/MOHD SHAZRUL FAZRY/25 SEPT./1034-SEPT.-2019-FEB-2020).

## Results

### Mucus collection and preparation

The epidermal mucus obtained from *A.* *testudineus* was scrapped to yield the mucus sample. The crude mucus content in the epidermal mucus of all selected healthy *A.* *testudineus* fish was 31.03 ± 0.21 mg/ml. Crude mucus that was obtained from the epidermal layer of *A.* *testudineus* was found to contain most of the basic biochemical components like proteins, carbohydrates, and lipids.

### SDS-PAGE protein fraction profile

**Figure 3:**
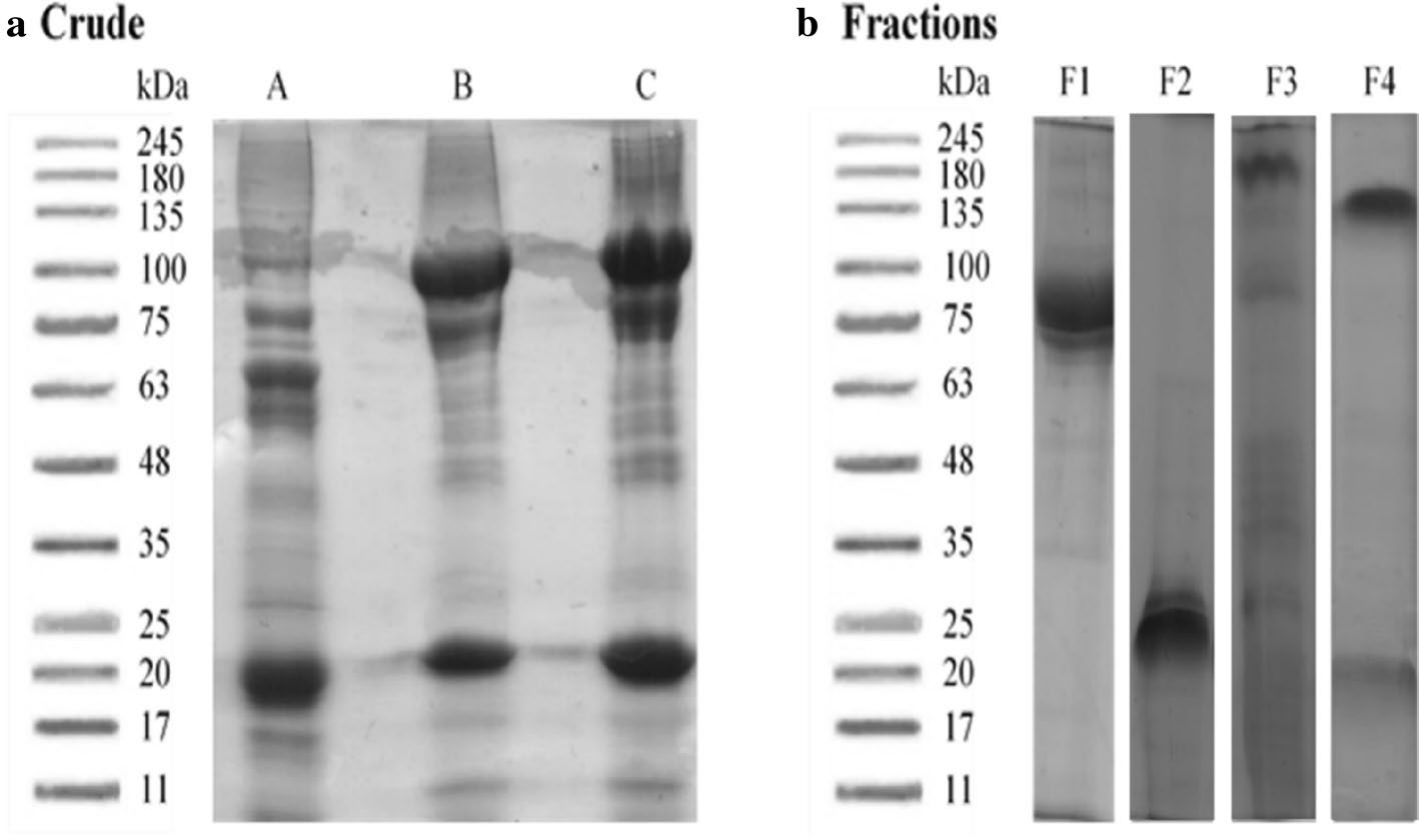
Protein profile for crude and fraction by 12% SDS PAGE from Sephadex G-25. (**a**) Crude mucus (AA) represents 1st replicate, (AB) represents 2nd replicate, (AC) represents 3rd replicate. (**b**) Protein fractions (F1) represents 1st fraction, (F2) represents 2nd fraction, (F3) represents 3rd fraction, (F4) represents 4th fraction. The figures are combination of different gels blot picture, and the original picture are shown in (Supplementary Figs. S1, S2, S3, S4, S5).

The protein profiles of the crude mucus protein samples of *A.* *testudineus* on SDS-PAGE gel showed proteins ranging from 245 kDa to less than 11 kDa (Fig. 3a). The crude mucus fractionated using Sephadex G-25 column. The protein fractions were collected, and the concentration of fraction measured using UV spectrometer at 280 nm^35^. The total protein content of the homogenate, dialyzed fractions were found to be 112, 138, 89, and 58 mg/ml, respectively. The protein profile for the fractions were analysed by 12% SDS-PAGE, which revealed a distinct protein of molecular weight ranging from 11 to 135 kDa (Fig. 3b). The molecular weight of fractions 1, 2, 3 and 4 were 75, 25, 80, and 135 kDa, respectively.

### Antimicrobial activity of protein fraction

**Figure 4:**
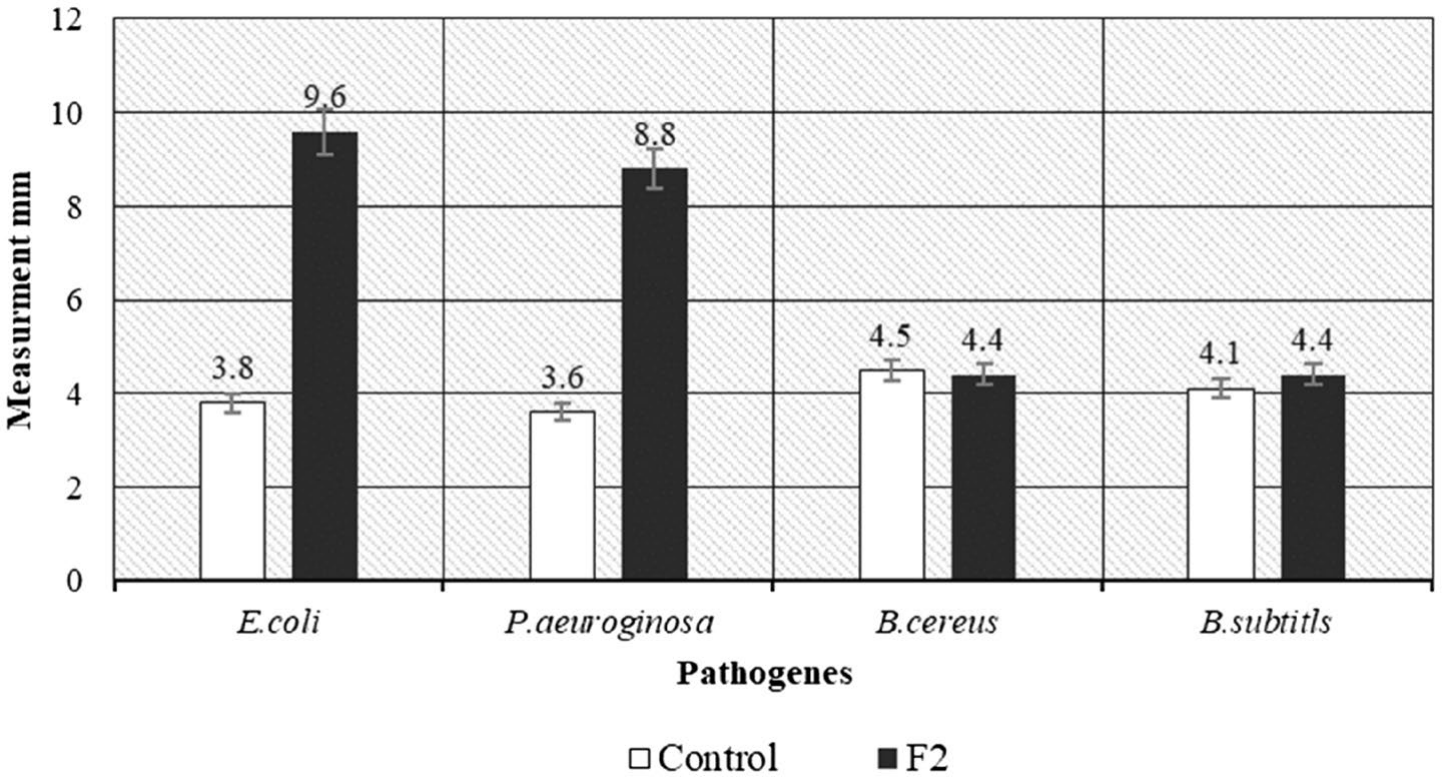
Antibacterial activity of F2 using disk diffusion method.

The cytotoxicity and antimicrobial effect of the protein fractions of *A.* *testudineus* were evaluated in-vitro to assess their activity. Among these fractions, only F2 show significant antimicrobial activity. This fraction showed higher antibacterial activities against the human pathogen (*E.* *coli,* *P.* *aeruginosa,* *B.* *subtilis,* *and* *B.* *cereus*) compared to the streptomycin as a control. The inhibition zones against *E.* *coli,* *P.* *aeruginosa,* *B.* *cereus,* *and* *B.* *subtilis* were 9.6 ± 0.12, 8.8 ± 0.20, 4.4 ± 0.62 and 4.1 ± 0.15 mm respectively (Fig. 4). These values are significantly higher compared to streptomycin (control) against *E-coli* (3.8 ± 0.18 mm) and *P*. *aeuroginosa* (3.6 ± 0.18 mm), but no significant different for *B.* *cereus* and *B.* *subtilis*. The correlation analysis using the bivariate analysis at 95% confidence level showed significant results (p < 0.01) between EM and streptomycin (control) of *E.* *coli* and *P.* *aeruginosa* pathogens.

### Cytotoxic effect of protein fraction

**Figure 5:**
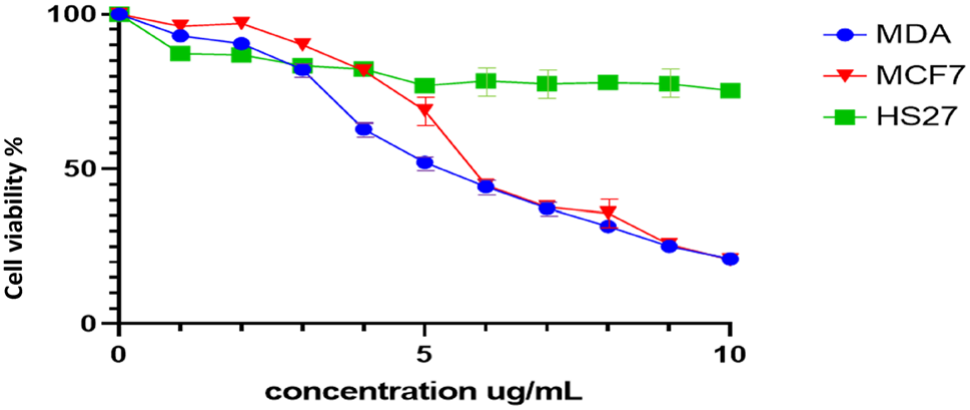
Cytotoxicity activity of F2 using MTT assay.

The cytotoxicity effect of F2 was assessed using MTT assay against human breast cancer cell line (MCF7), human adenocarcinoma cell line (MDA-MB-231), and normal cell, human new-born for skin fibroblast (HS27). These cells were treated with F2 at different concentration 1–10 μg/ml for 24 h and 48 h (Fig. 5). This treatment showed that the percentage of cell viability gradually decreased as F2 concentration increased. The IC_50_ value of those cells (MCF7, and MDA-MB-231) treated for 48 h with F2 were 5.02 ± 0.4 μg/ml and 4.97 ± 0.25 μg/ml respectively. This treatment shows no significant effect on normal cells (new-born skin fibroblast). The cytotoxicity results showed that F2 has cytotoxicity effects against cancer cells while did not show any effects against normal (healthy) human cells.

### Cell cycle profile

**Figure 6:**
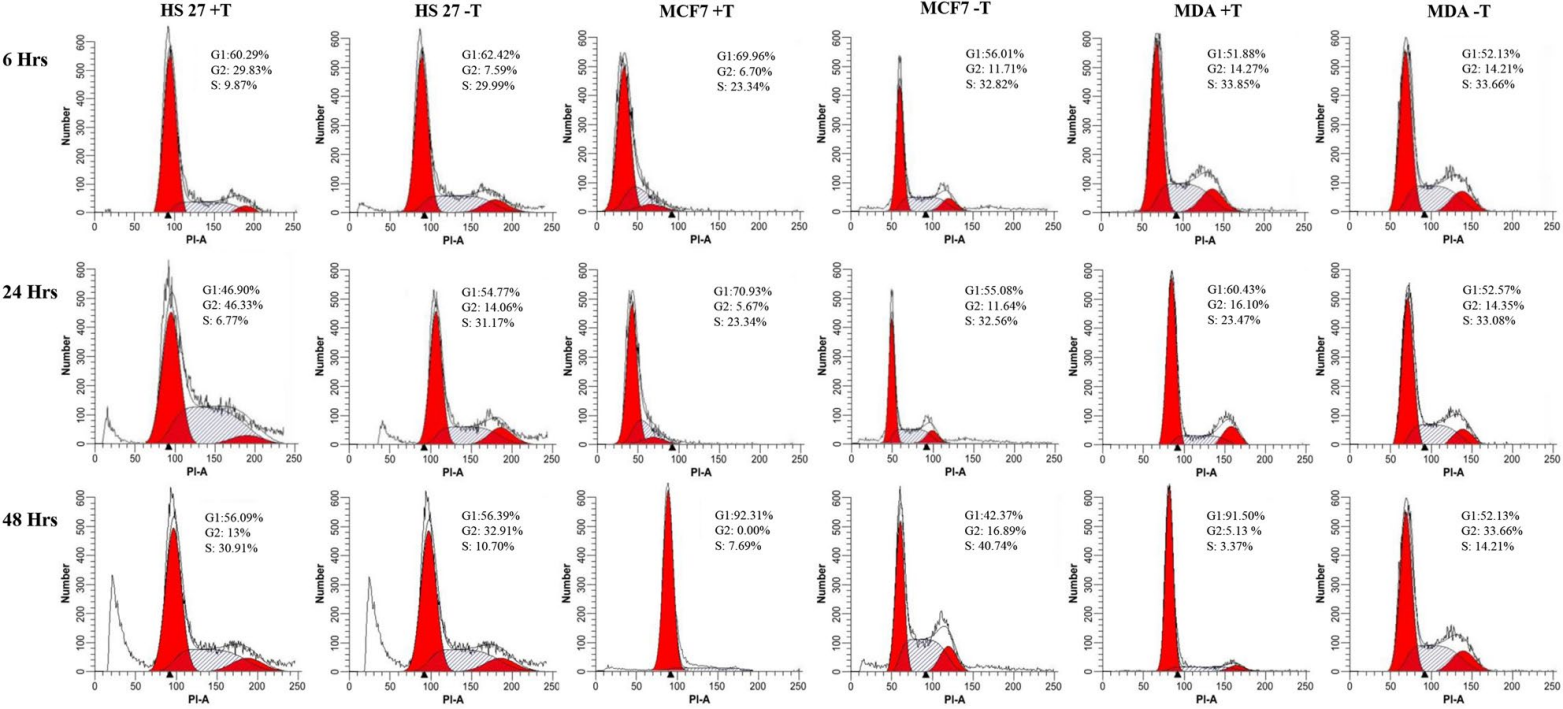
Cell cycle profile for cells after treatment with F2. (+ T) treated sample, (− T) untreated sample.

Fluorescence-activated cell sorting (FACS) is a specialized type of flow cytometry in which utilizes light to count and profile cells in a heterogeneous fluid mixture. In this study, the cell cycle treated with F2 were observed for 48 h. The cancer cell cycle of MCF7 and MDA-MB-231 was observed at four-time points (6 h, 12 h, 24 h, and 48 h) and compared with the normal cell lines HS27 at the same conditions (Fig. 6). Compared to the untreated, F2 arrested the growth of MCF7 and MDA-MB-231 cells at the G0/1 phase. F2 did not show any effects on the cell cycle of HS27. In this study, HS27 has been used to study and evaluate the effects of F2 on normal cells. F2 arrested the growth of MCF7 and MDA-MB-231 cells at the G0/1 phase but not HS27. The arrest of cells growth at the G0/1 phase is very important as it will block the cancer cells from entering the S-phase resulting in DNA damage^19,34^.

### DNA fragmentation assay

**Figure 7:**
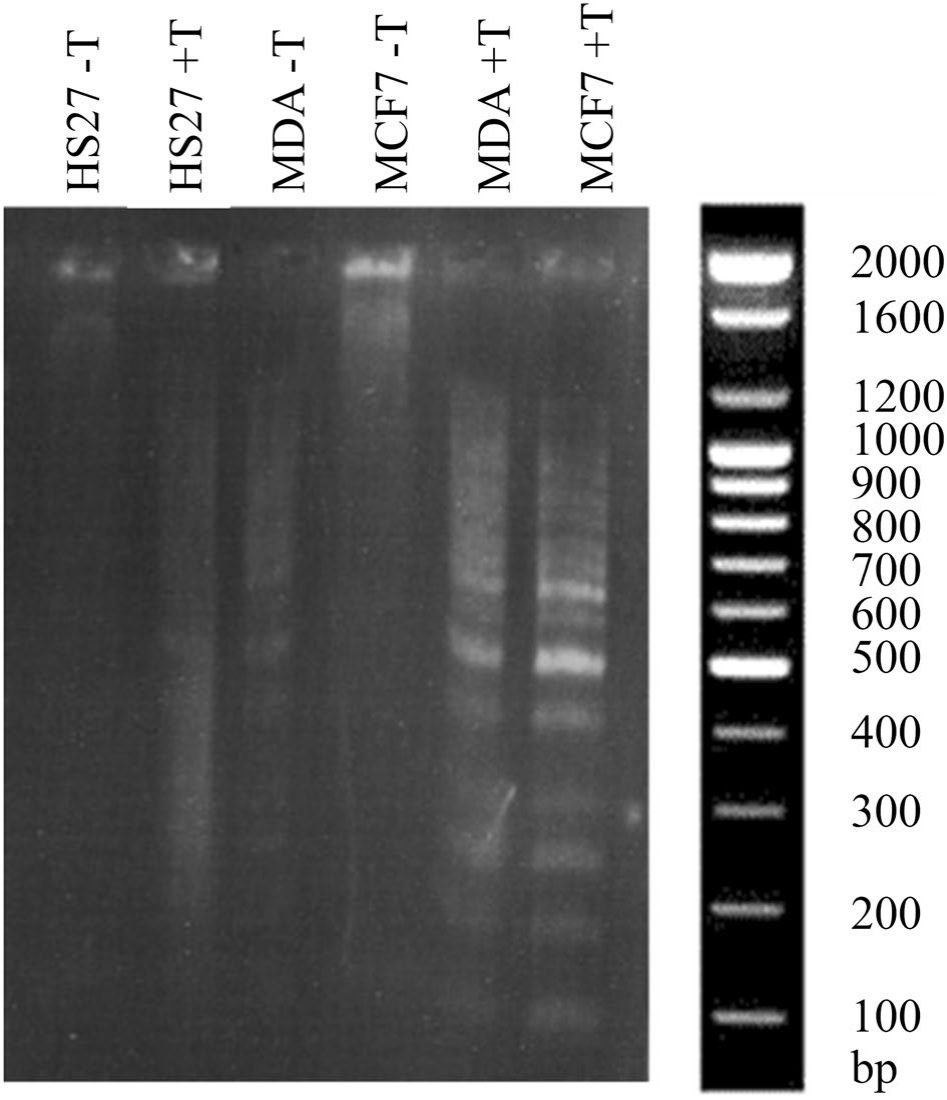
DNA fragmentation assay for cells after treatment with F2. (HS27-T) Represented non-treated HS27, (HS27 + T) Represented treated HS27, (MDA-T) Represented non-treated MDA-MB-231, (MDA + T) Represented treated MDA-MB-231, (MCF7-T) Represented non-treated MCF7, (MCF7 + T) Represented treated MCF7.

To detect apoptosis, this study employed DNA fragmentation assay by using 1.2% agarose gel electrophoresis on the DNA extracted from MCF7, MDA-MB-231, and HS27 cells that was treated with 5 μg/ml F2 for 48 h incubation. It was found that the DNA obtained from control samples was intact and represented as a prominent single band on the gel. On the contrary, the electrophoretic DNA obtained from cell lines treated with F2 has a few fragments. Results showed that breast cancer (MDA-MB-231 and MCF7) cell lines treated with 5 μg/ml of the F2 samples showed higher fragmentation than HS27 as control (Fig. 7).

### AMPs selection and synthesis

This study found that F2 was able to reduce cell viability. These results led to the hypothesis that peptides or proteins within the F2 could have different cytotoxic mechanisms against the cells. Modification of these peptides or proteins could enhance specificity to the MCF-7 and MDA-MB-231 cells. Thus, F2 was selected for peptidomics analysis. This fraction was then further separated by SDS-Page, digested, and directly subjected to the LC–MS/MS analysis.

Four bioinformatics programs including ADP3, AMPfun, ADAM and AntiBP2 were used to predict putative AMPs from the mass spectrometric-detected peptides. Seven putative AMPs were predicted from F2 which having positive prediction scores. Most of the predicted putative AMPs have a molecular weight less than 10 kDa with length ranging from 17 to 25 amino acids. Analysis of physicochemical properties of these 7 putative AMPs by ADP3 showed a higher average score of hydrophobicity from 47 to 12% and net charge from − 2 to + 2, as shown in Table 1. Then the physiochemically properties and N-terminus, C-terminus, and NC-terminus for those peptides were referenced in ADAM and AntiBP2 data bases. Two peptides were identified by AMPfun having good properties to function as antibacterial and anticancer (Table 1). Both have sequence “THPPTTTTTTTTTTTTTAAPATTT”, and “TGIATSGLATFTLHTGSLAPAT”. These two sequences were used for production of synthetic AMP and coded as AtMP1 and AtMP2 respectively.

### Antimicrobial activity of AMPs

**Figure 8:**
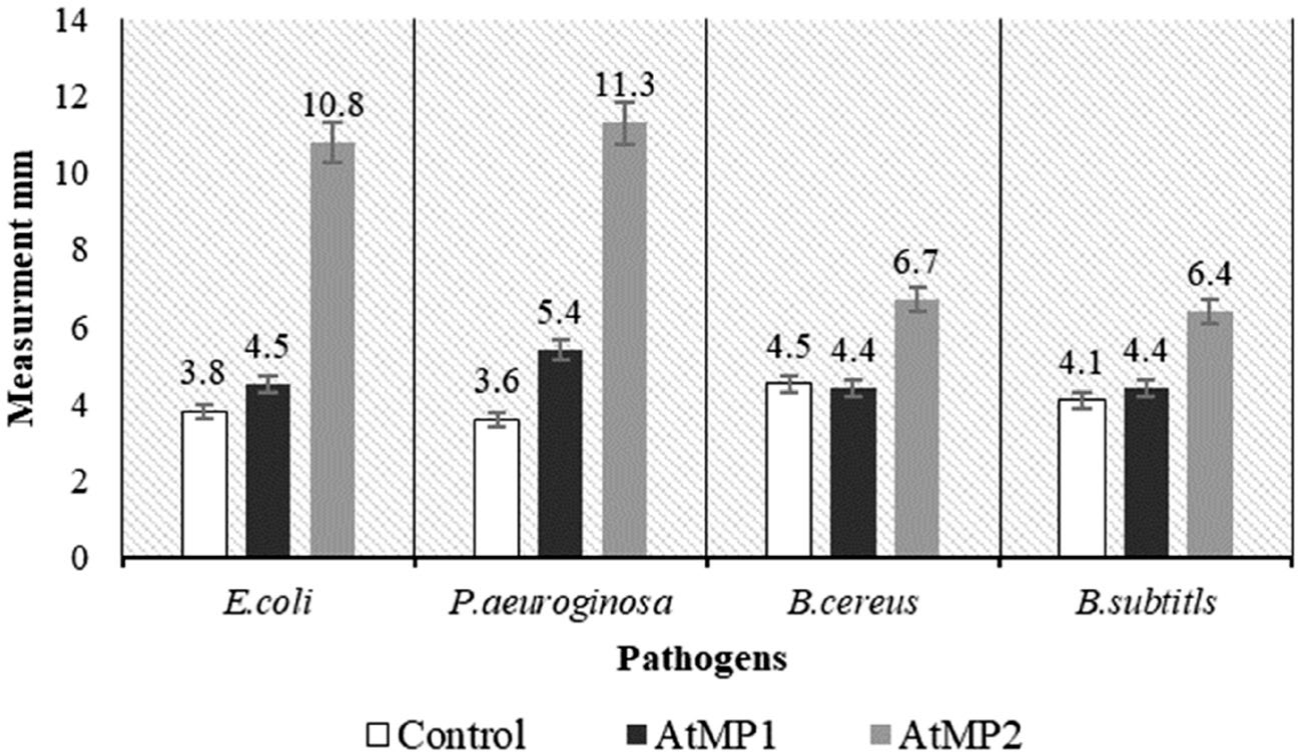
Antibacterial activity of peptides using disk diffusion method. The data were expressed as the mean ± standard deviation (error bar) for three replicates.

The disc diffusion method was used to test the antimicrobial activity of AtMP1 and AtMP2 against the human pathogens (*E.* *coli*, *P.* *aeruginosa*, *B.* *subtilis*, and *B.* *cereus*) while compared to streptomycin as control. The diameter of inhibition zone formed were presented in Fig. 8. It was found AtMP2 significantly have higher antimicrobial activity compared to AtMP1 and commercial antibiotic, streptomycin. The correlation analysis using the bivariate analysis at 95% confidence level showed significant results (p < 0.01) between AtMP2, AtMP1 and the control. The antimicrobial activity of AtMP2 is even higher compared to their parent fraction, F2 (Fig. 4).

### Cytotoxic effect of AMPs

**Figure 9:**
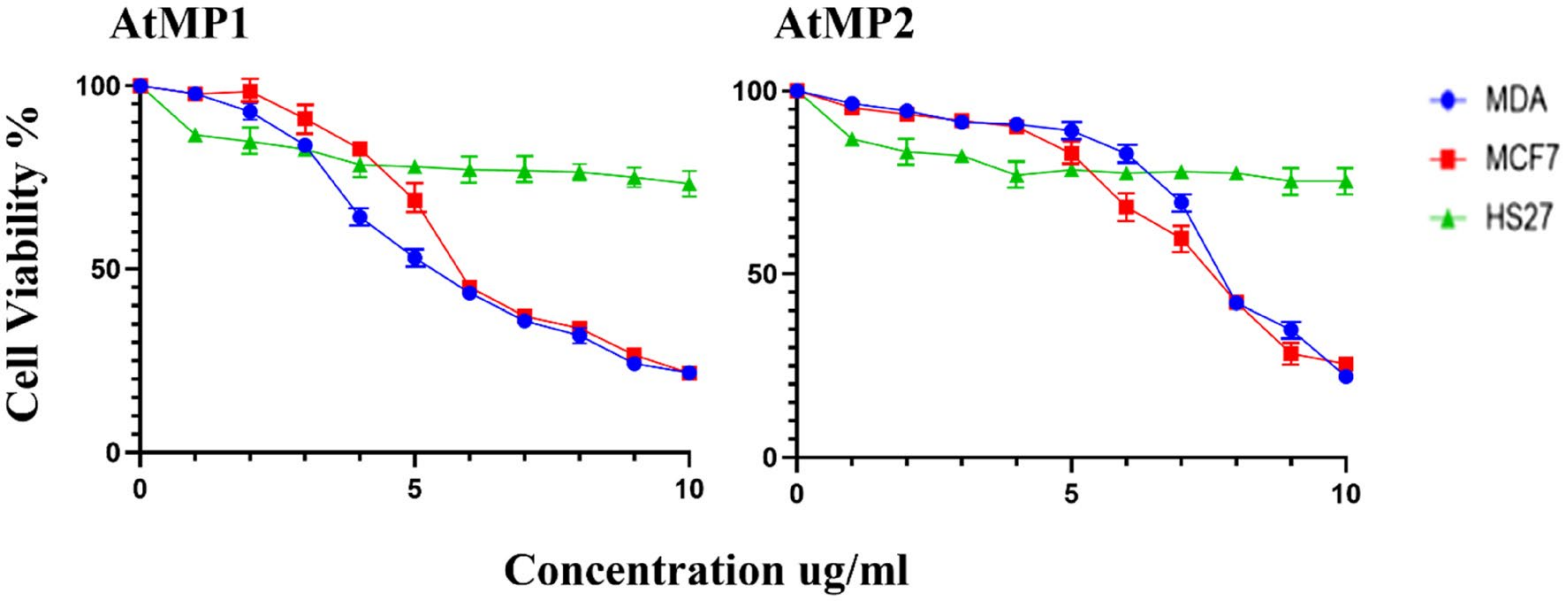
Cytotoxicity effect of synthesis AtMP1 and AtMP2 on cancer MDA-MB-231 and MCF7 cell line and HS27 normal cell lines. the cytotoxicity calculated and the results show the treatment was cytotoxic cancer cell line after 48 h. However, the treatment didn’t show effects against normal cell line (HS27). The graphs are representing cell viability (%) by measuring the absorbance of optical density (590 nm) with a microplate reader. Data are the mean ± SD of triplicate determinations. (Left) AtMP2. (Right) AtMP1.

Peptide cytotoxicity effect against the MCF-7, MDA-MB-231 and HS27 were analysed by MTT assay for concentration range from 1 to 10 μg/ml. It was found increased in concentration of peptides significantly reduced the viability of all cells. However, both peptides didn't show a significant effect against HS27 cell-line (Fig. 9)**.** The IC_50_ of the AtMP1 against MCF7 and MDA-MB-231 were 8.25 ± 0.14 and 9.35 ± 0.25 μg/ml respective. AtMP2 show lower IC50 value which were 5.89 ± 0.14 and 6.97 ± 0.24 μg/ml against MCF7 and MDA-MB-231 respectively.

### Annexin V-FITC apoptosis assay

**Figure 10:**
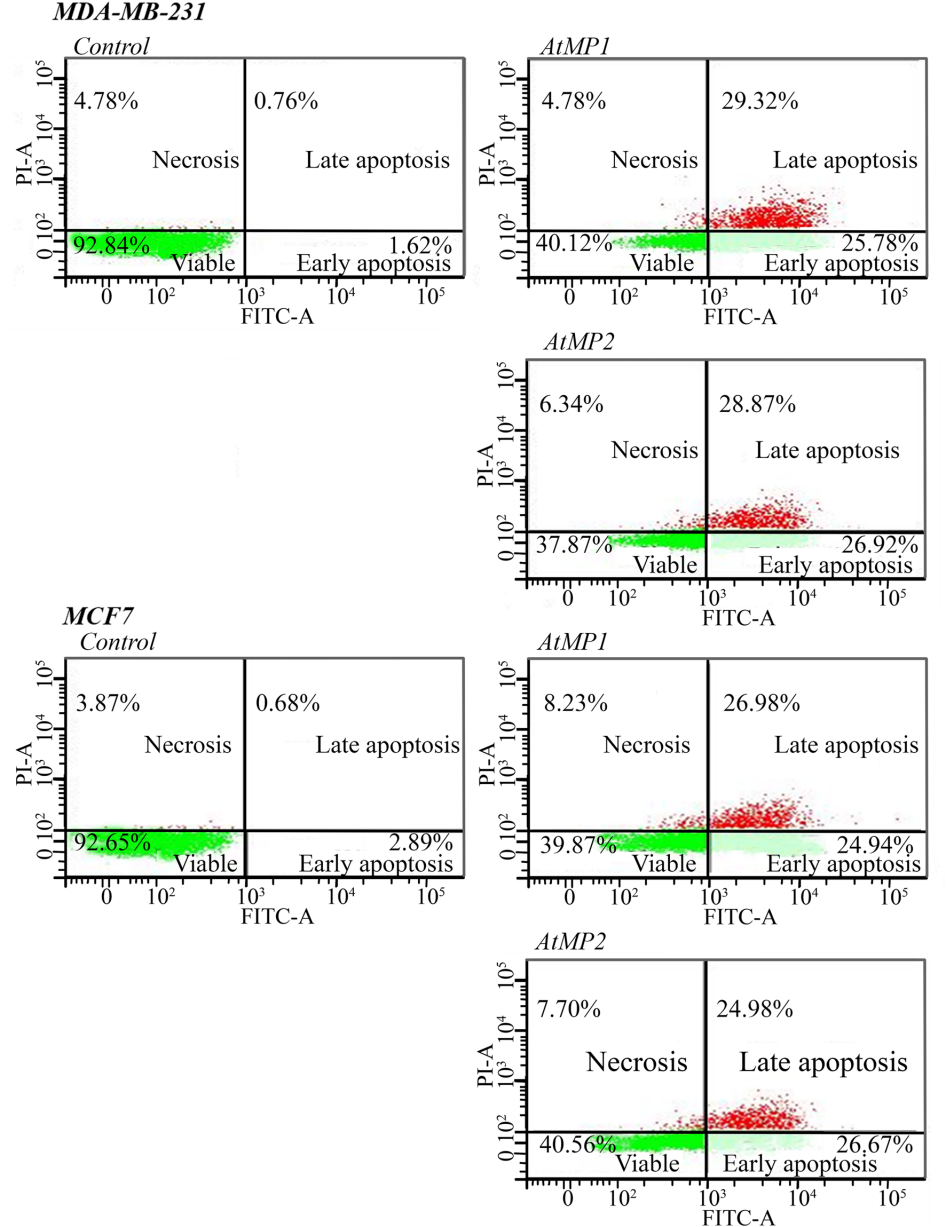
Apoptosis detection results by Anexien V FTIC-A assay.

To detect the apoptosis of AtMP1 and AtMP2 in MDA-MB-231 and MCF7 cell-lines, Annexin V-FITC method was applied. This study used untreated breast cancer cell lines MDA-MB-231 and MCF-7 as a control to compare with the treated cell lines to check the effect of AtMP1 and AtMP2 on breast cancer cell lines. Dot-plot graphs Fig. 10, the lower right quadrant refers to early phase apoptotic cells, the upper right quadrant refers to late phase apoptosis, the upper left quadrant refers to necrotic cells, and the lower left quadrant refers to viable cells. These results indicated that at the early apoptosis stage, the mitochondrial membrane was disturbed, and the phosphatidylserine appeared on the cell surface, which linked to Annexin V. This event is considered as one of the early apoptosis markers. Meanwhile, the nuclear membrane was lysed at the late-stage apoptosis, and the stain entered the nucleus. AtMP1 and AtMP2 showed as effective molecules that act against cancer cells either via the membranolytic pathway or by rupturing the mitochondrial membrane. As shown in Fig. 10 the early apoptotic cell populations of MCF-7 for the AtMP1 and AtMP2 at 48 h was 24.94 ± 0.53 and 26.67 ± 0.43, and the late apoptotic cells of MCF7 were 26.98 ± 0.54 and 24.98 ± 0.56, respectively. Untreated MCF-7 cells didn’t show any apoptosis level compared to treated cells. For MDA-MB-231 early apoptotic cell populations for the AtMP1 and AtMP2 at 48 h was 25.78 ± 0.26 and 26.92 ± 0.12, and the late apoptotic was 29.32 ± 0.31 and 28.87 ± 0.15, respectively. Untreated MDA-MB-231 cells didn’t show any apoptosis level compared to treated cells.

### Gene expression profiler RT^2^ PCR array

**Figure 11:**
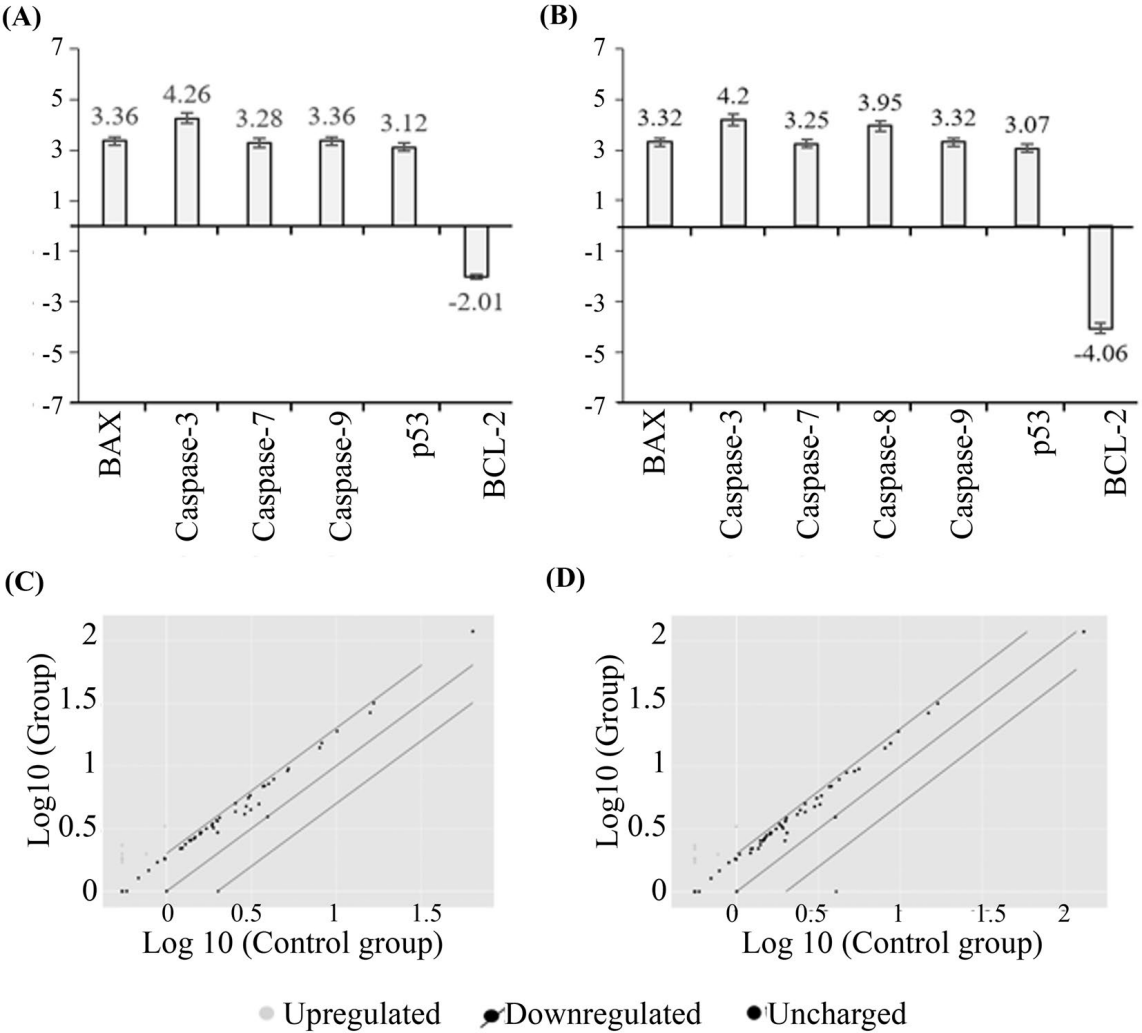
RT^2^ Profiler PCR Array (PAHS-012ZA) for MDA-MB-231 cell lines. (**A**) The fold change values for cancer cells treated with the AtMP1, (**B**) fold change values for cancer cells treated with the AtMP2. (**C**) The Scatter Plot compares every gene's normalized expression on the PCR Array for cancer cells treated with the AtMP1, (**D**) the Scatter Plot compares the normalized expression of every gene on the PCR Array for cancer cells treated with the AtMP2. The results analysis by using Gene Globe Data Analysis. Genes showed less than 1.5-fold change were neglected. The full list of genes was mentioned in supplementary figures file (Supplementary Tables S1, S2).

**Figure 12:**
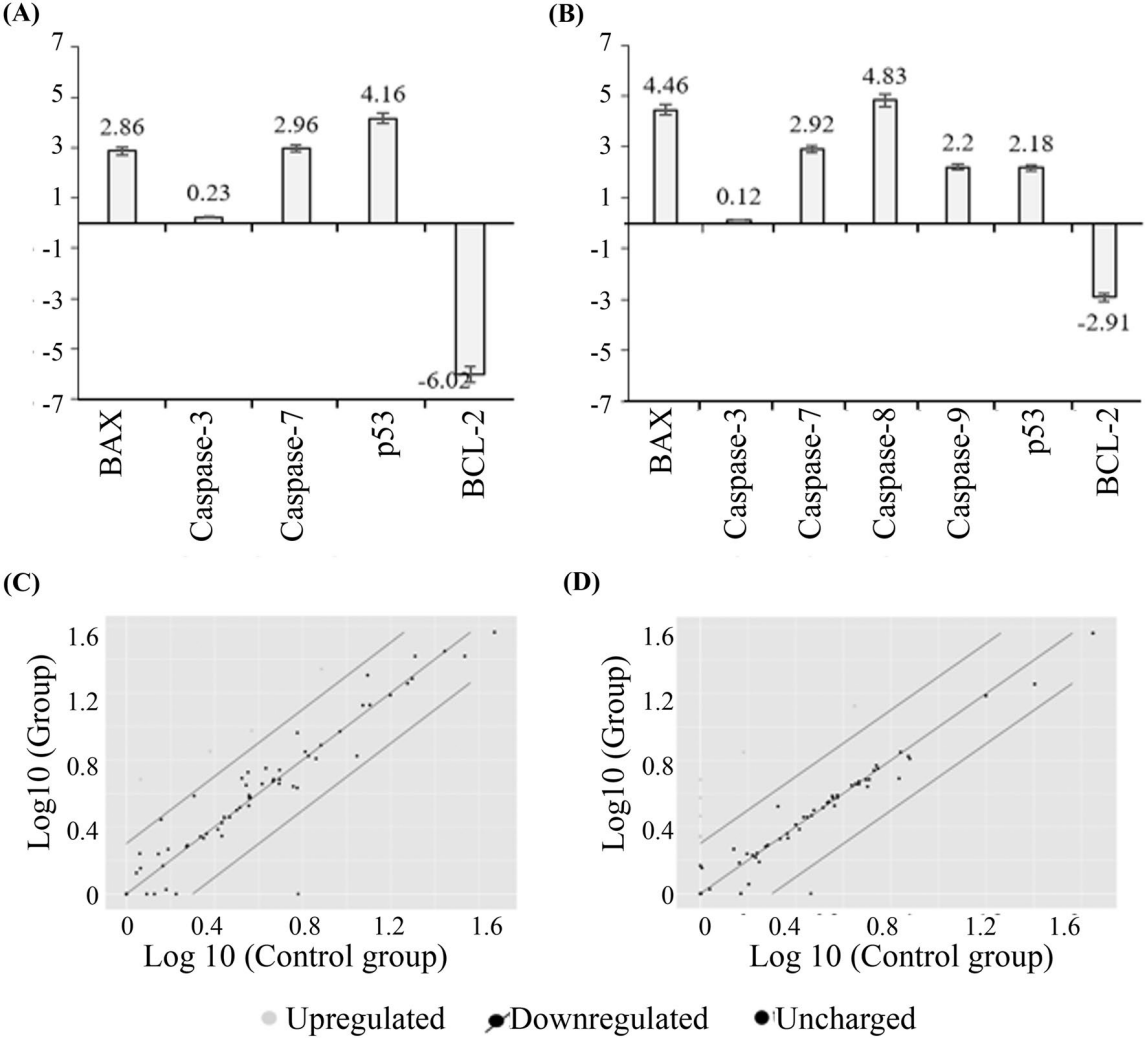
RT^2^ Profiler PCR Array (PAHS-012ZA) for MCF7 cell lines. (**A**) the fold change values for cancer cells treated with the AtMP1, (**B**) fold change values for cancer cells treated with the AtMP2. (**C**) The Scatter Plot compares every gene's normalized expression on the PCR Array for cancer cells treated with the AtMP1, (**D**) the Scatter Plot compares the normalized expression of every gene on the PCR Array for cancer cells treated with the AtMP2. The results analysis by using Gene Globe Data Analysis Centre. Genes showed less than 1.5-fold change were neglected. The full list of genes was mentioned in supplementary figures file (Supplementary Tables S3, S4).

In order to detect the apoptosis pathway of the used cancer cells and the responsible gene regulating the apoptosis induced by the experimental treatments, the human apoptosis cancer RT^2^ Profiler PCR Array (PAHS-012ZA) was used. This test included 84 essential gene-regulated human apoptosis. These genes relate to a specific pathway, which contains proteins (genes) that can contribute to one or more processes, including cell cycle monitoring, angiogenesis, and apoptosis. The details on fold change are summarised in Figs. 11 and 12.

In this test, many genes were observed to be upregulated or downregulated. However, these genes showed less than 1.5-fold change; therefore, it was neglected. The AtMP1 significantly upregulated the expression of four genes in the MCF7 cancer cell, including BAX, caspase-3, caspase-7, and p53, and downregulated the expression of BCL-2. For the MDA-MB-231 cancer cells, the AtMP1enhanced the five gene expressions, including BAX, caspase-3, caspase-7, caspase-9, and p53, and diminished the expression of the BCL-2 gene. When the AtMP2 was used to treat MCF7 cancer cells, three genes were upregulated, including BAX, caspase-7, and p53, and the BCL-2 gene was downregulated (> sixfold). However, the cancer cell MCF7 treated with the AtMP1 and AtMP2 did not show any significant regulation on caspase-3, yet MCF7 led to apoptosis induction (this might be due to the activation of caspase-7).

In the MDA-MB-231 cancer cell, the AtMP2 significantly upregulated the five genes' expression (BAX, caspase-7, caspase-8, caspase-9, and p53), and diminished the BCL-2 gene. These results showed that the AtMP1 and AtMP2 could enhance the downregulation of the anti-apoptotic BCL-2 gene. Furthermore, they promoted the upregulation of pro-apoptotic genes, such as BAX; tumor suppressor genes, such as p53; and the executioner caspase family, such as caspase-3, caspase-7, caspase-8, and caspase-9, simultaneously.

### Bioinformatic prediction of protein-peptide docking

**Figure 13:**
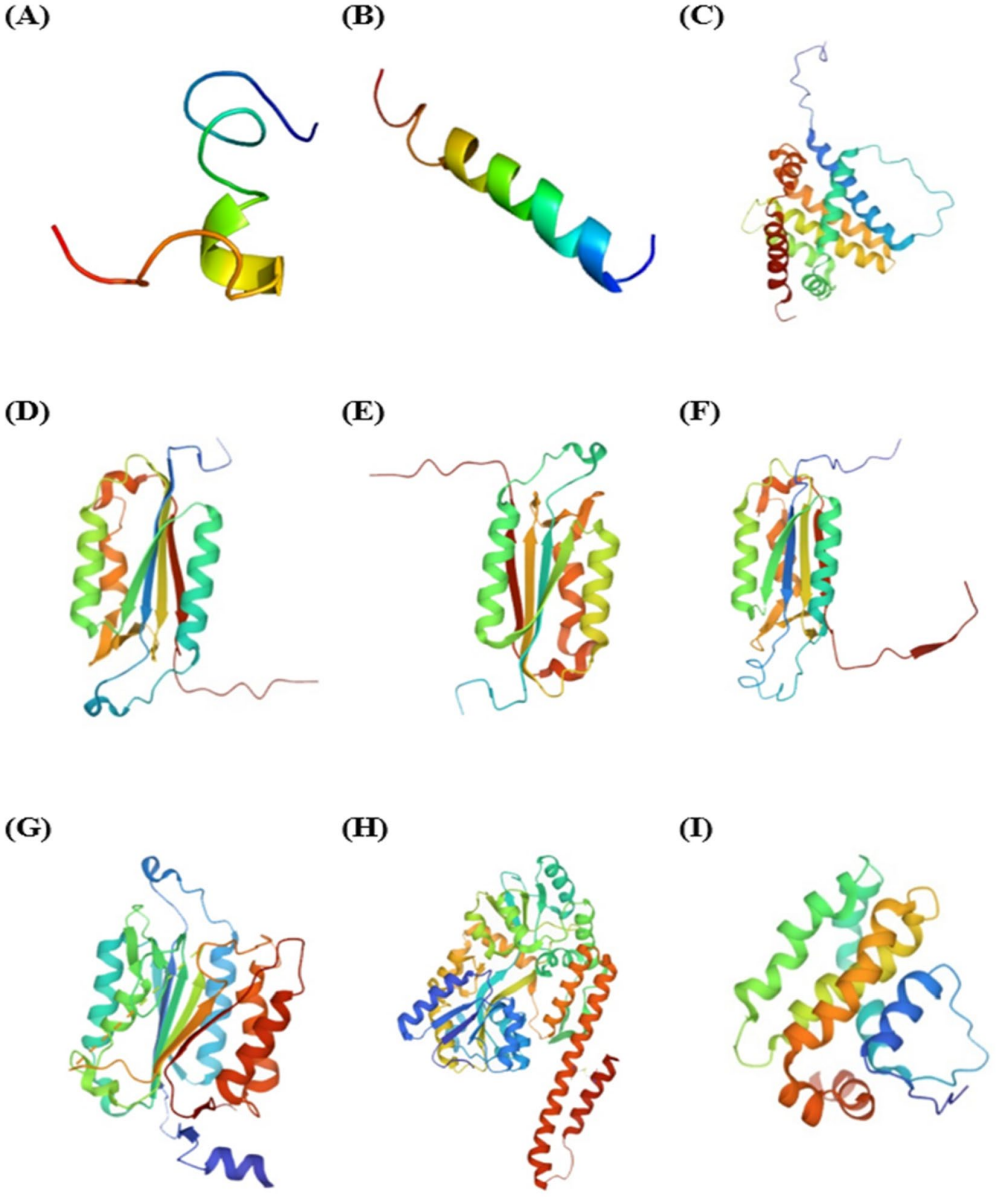
3D model structure for peptides and proteins by using QuickDBD. (**A**) AtMP1, (**B**) AtMP2, (**C**) BAX, (**D**) caspase-3, (**E**) caspase-7, (**F**) caspace-8, (**G**) caspase-9, (**H**) p53, and (**I**) BCL-2.

After gene expression identification, this study aimed to specify the structure of the selected two peptides, the peptides' binding site and the interacted seven genes, and the interaction model. A set of five databases and software was used, including QuickDBD^40^, RCSB PDB^41^, HPEPDOCK^42^, ZDOCK^39^, and the ligand and protein files were prepared through BIOVIA DSV software^43^ to detect the blind docking method of peptides to proteins. The sequence of the two selected peptides (Table 1) was submitted to QuickDBD using the Fasta format^40^. The QuickDBD showed 10 models for each peptide structure. Among these models, the top-score models were selected based on the Z-score (− 239.700 for the AtMP1 and -173.695 for the AtMP2 as shown in Fig. 13. The RCSB PDB protein data bank (was utilised to detect the sequence length, chain, and active site of the interacted proteins^41^.

**Figure 14:**
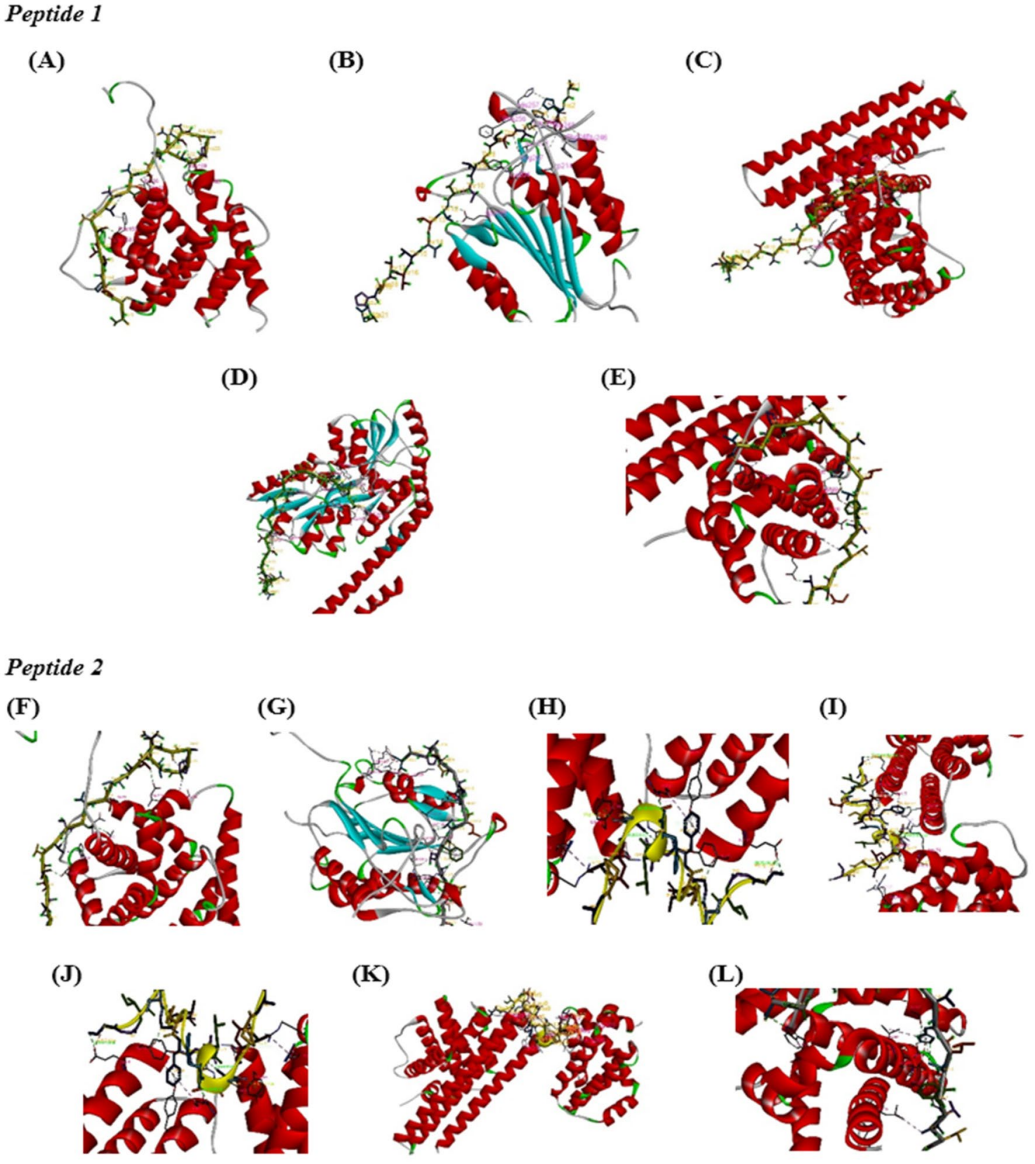
Peptide-protein docking 3D model by using HPEDOCK. (**A**) AtMP1-BAX docking model, (**B**) AtMP1-caspase-3 docking model, (**C**) AtMP1-caspase-9 docking model, (**D**) AtMP1-p53 docking model, (**E**) AtMP1-BCL2 docking model, (**F**) AtMP2-BAX docking model, (**G**) AtMP2-caspase-3 docking model, (**H**) AtMP2-caspase-7 docking model, (**I**) AtMP2-caspase-8 docking model, (**J**) AtMP2-caspase-9 docking model, (**K**) AtMP2-p53 docking model, (**L**) AtMP2-BCL2 docking model. The results showed the interaction site between the peptides and the selected cell proteins (Original image are shown in Supplementary Figs. S6, S7, S8, S9, S10, S11, S12, S13, S14, S15, S16, S17.

After that, the model of each peptide was submitted sequentially with each protein to the HPEPDOCK database^42^, which led to identifying each peptide's binding site with the proteins. HPEDOCK also aided in identifying the type of binding bonds and the interaction model between the peptide and proteins (peptide-protein docking) (Fig. 14).

Figure 14 indicates that the AtMP2 can be linked to BAX (THr22-Glu123, THr1-Tyr204, Glu3-Phe256, His13-Arg207, Pro20-Asn208, Ala9-Trp214/Phe247/Glu246, Lys4-His257/Glu248/Ser249) and caspase-3 (Ile3-Phe252, Ser6-Ser209, Leu8-Arg207, Ala9-Trp206, THr10-Ser205/Cys163, Phe11-His121, THr12-Gly122/Glu123, His14-Pro133/Glu123, THr22-Cys138/Lys137/Asn141/Arg147/Arg144). The AtMP2 interacted with caspase-7 (Leu8-His121/Tyr204, Phe11-Lys256/Ser251, His14-Asp253/Ser251, Gly16-Ser209) and caspase-8 (THr1-Glu115, Gly7-Tyr116, Phe11-Gly117/Tyr29/Arg33, His14-Arg33, Gly16-Tyr175, Leu18-Asp172, Ala19-Arg300).

Then, the AtMP2 was linked to caspase-9 (Ala4-Pro198, THr5-Glu180, Ala9-Leu179, Phe11-Asp296, His14-Ser247/Val297/Ser293/Asp296, Ala21-Trp305, THr22-Trp305), p53 (THr1- Mel330/Trp62/Asp65, His13-Trp62/Lys15/Trp230/Asp14, Pro20-Phe156/Tyr155/ Trp230/Asn12/Asp14/Lys15, Lys17-Glu38, Ala4-Asp55/Lys1, Ala19-Lys1). In addition, the AtMP2 was inserted into BCL-2 (Ala4-Pro189, THr5-Glu180, Ala9-Leu179, THr10-Glu292, Phe11-Asp296, His14-Ser247, Val297, Asp296, Ser293, THr22-Trp305).

**Figure 15:**
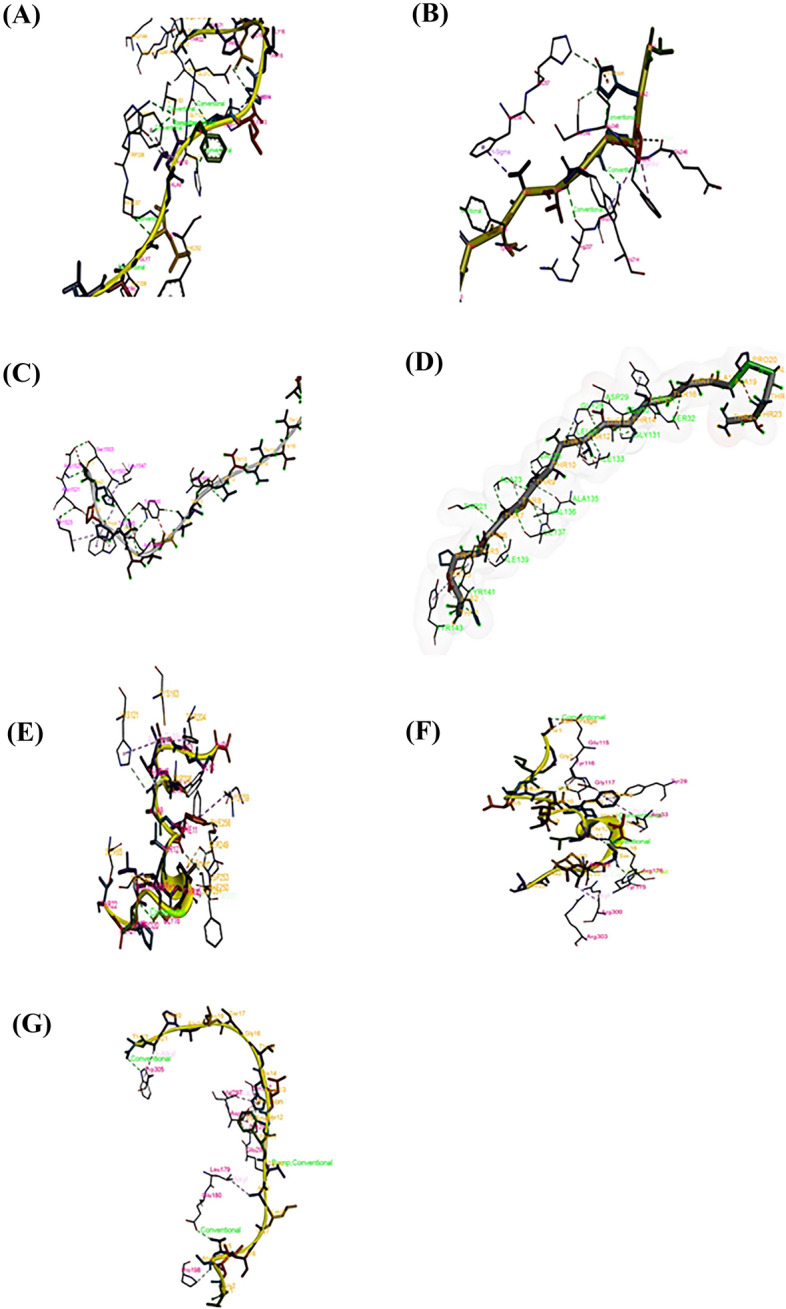
Peptide-protein docking 3D model by using ZDOCK. (**A**) AtMP2-BAX docking model, (**B**) AtMP2-caspase-3 docking model, (**C**) AtMP2-caspase-7 docking model, (**D**) AtMP2-Caspase8 docking model, (**E**) AtMP2-Caspase9 docking model, (**F**) AtMP2-P53 docking model, (**G**) AtMP2-BCL-2 docking model. The results showed the interaction site between the peptides and the selected cell proteins (original images are shown in Supplementary Figures S25, 26, 27, 28, 29, 30, 31).

For reliability, the ZDOCK database^39^ was utilised to confirm the interaction model of each peptide that interacted with the proteins. Finally, the predicted models of the peptide-protein docking were visualised and modified using BIOVIA DSV software^43^ as shown in Fig. 15. These databases provided useful tools for investigating peptide-protein docking, modelling, and reconstruction. Notably, the findings obtained from these databases proved the validity of gene expression results, in which the two peptides extracted from *A.* *testudineus* could modulate the expression of the genes (BAX, caspase-3, caspase-7, caspase-8, caspase-9, p53, and BCL-2). All the data bases websites links and date of access were provided in Supplementary Table S5.

### Immunoprecipitation pull-don assay

**Figure 16:**
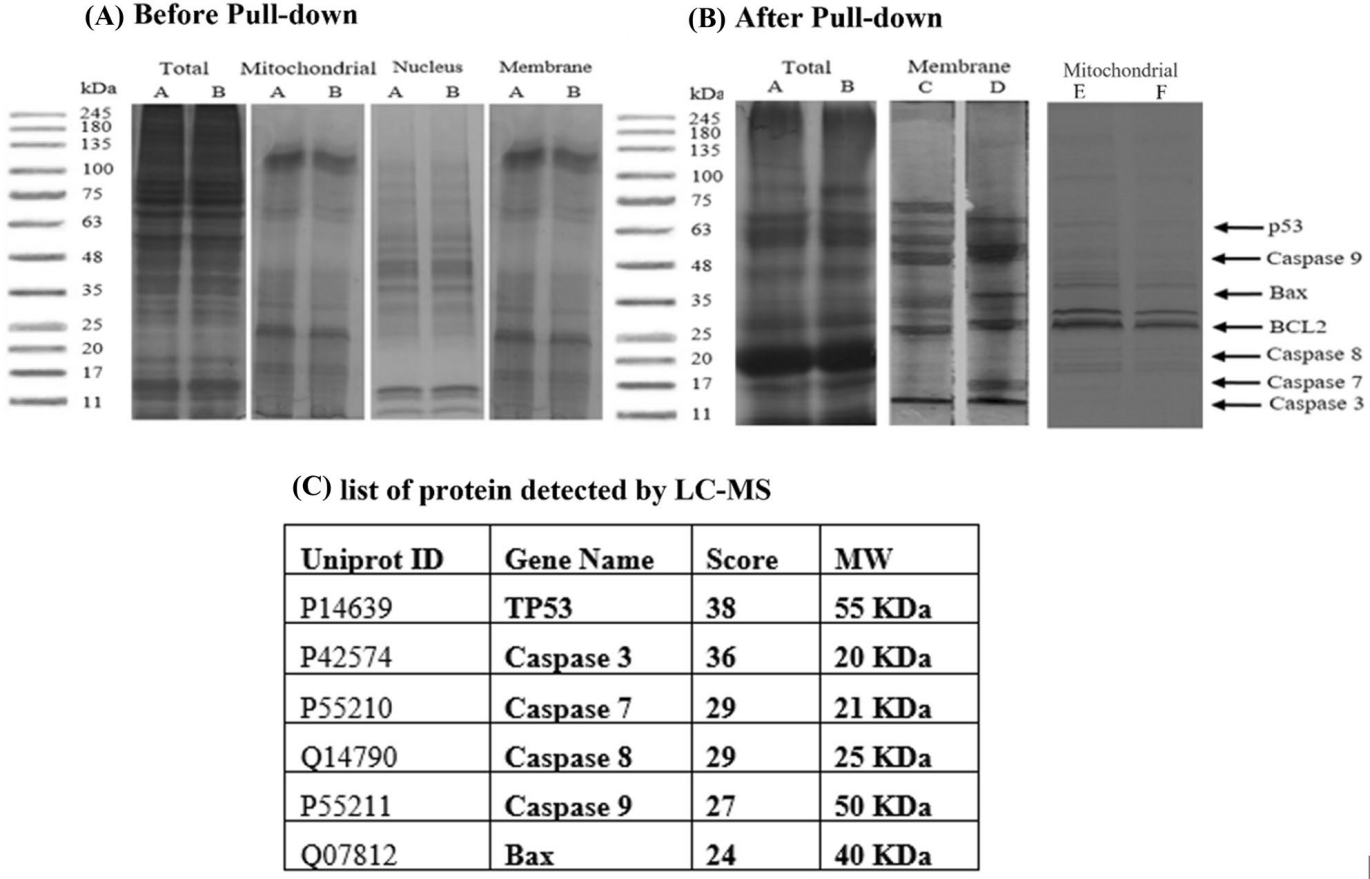
Peptide-protein immunoprecipitation analysis. (**A**) Total, membrane, and mitochondrial cell lysate before pulldown. (BA) total proteins linked to AtMP1 (BA) and (BB) total proteins linked to AtMP2 (BC) membrane proteins linked to AtMP1, and (BD) membrane proteins linked to AtMP2, and (BE) mitochondrial proteins linked to AtMP1, and (BF) mitochondrial proteins linked to AtMP1. (**C**) The list of cell proteins detected in LC–MS. (Gel blot figures are combination of different gels. The original pictures are shown in (Supplementary Figs. S18, S19, S20, S21, S22, S23, S24).

Immunoprecipitation was carried out of 1.5 mg total cell lysates extracted from MCF7 and MDA-MB-231 cells using agarose beads to study the protein-peptide interaction network. The cell lysates (the total, mitochondrial, membrane, and nucleus) of non-treated cell lines were extracted and run in SDS-PAGE along with marker size ranging from 11 to 245 kDa. Results show the present of the high and low molecular weight (MW) bands in the range of 11–245 kDa (Fig. 16A). Then, both breast cancer cell lines (MCF7 and MDA-MB-231) were treated with biotinylated peptides, AtMP1 and AtMP2, and incubated for 48 h. Both peptides treated cell lysates were extracted and precleared with the same material used in immunoprecipitation to reduce nonspecific binding proteins and run in SDS-PAGE along with same marker size (11–245 kDa) (Fig. 16B).

The specific possible bands of apoptosis related proteins; caspase-3 (20 kDa), caspase-7 (21 kDa), caspase-8 (25 kDa), caspase-9 (50 kDa), p53 (55 kDa), and Bax (40 kDa) (Fig. 16B), were excised based on their MW range, digested, and analyzed by Nano liquid chromatography-tandem mass spectrometry (Gel-Nano LC–MS/MS) as presented in Supplementary Table S6.

Subsequently, the LC–MS peak list files were used to query NCBI, Swiss-Port, and Mascot 2.0 databases were applied for protein detection and identification. Only significant hits as defined by Mascot probability analysis were considered. Peptide hits were accepted when the ion score exceeded a value of 20. Protein hits required at least one peptide hit exceeding a peptide score of 20. Protein identifications were accepted with a statistically significant Mascot protein search score ≥ of 20 which corresponds to an error probability of P < 0.05. The apoptotic proteins identification with the adequate score was selected. The specific apoptosis proteins were identified via using LC–MS and NCBI, Swiss-Port, and Mascot 2.0 databases.

Overall, the combined LC–MS data resulted in the identification of 55 distinct protein species. A total of 55 proteins were identified and arranged in descending order based on their Mascot protein search score^27,55^ (Supplementary Table S6). Seven apoptotic proteins (p53, BCL-2, BAX, Caspase-3, Caspase-7, Caspase-8, Caspase-9) were found in the list of 55 proteins (Fig. 16C).

## Discussion and conclusion

As far as we are aware, the present study is the first study to examine the effect of *A.* *testudineus* fish skin mucus on breast cancer and normal cell line. Therefore, the current study examined the effect and role of the crude and four fractions of *A.* *testudineus* mucus on breast cancer cell line namely MCF7 and MDA-MB-231 in comparison to human skin fibroblast (HS27) by using the MTT assay. Disc diffusion method also used to examine the antimicrobial effect of crude and four fractions of *A.* *testudineus* mucus against four human pathogens. Consistent with this goal, the current study found that the F2 of *A.* *testudineus* skin mucus inhibited the proliferative of breast cancer cell line MCF7 and MDA-MB-231 through inducing cell apoptosis. Therefore, the F2 blasted against a database of antimicrobial peptides to screen its antimicrobial and anticancer properties. This proved that the F2 contained seven antimicrobial peptides, two of them have both antimicrobial and anticancer possible activity; the AtMP1 sequence was THPPTTTTTTTTTTTTTAAPATTT, and the AtMP2 sequence was TGIATSGLATFTLHTGSLAPAT. The IC_50_ of the AtMP1 against MCF7 and MDA-MB-231 were 8.25 ± 0.14 and 9.35 ± 0.25 μg/ml, and the IC_50_ of the AtMP2 against MCF7 and MDA-MB-231 were 5.89 ± 0.14 and 6.97 ± 0.24 μg/ml. Also, the inhibition zone of the pathogens (*E.* *coli*, *P.* *aeruginosa*, *B.* *cereus*, and *B.* *subtilis*) treated with the AtMP1 was 4.5 ± 0.11, 5.4 ± 0.24, 4.4 ± 0.18, and 4.4 ± 0.12 mm, respectively. The inhibition zone of the mentioned pathogens treated with the AtMP2 was 10.8 ± 0.24, 11.3 ± 0.23, 6.7 ± 0.17, and 6.4 ± 0.21 mm, respectively (Fig. 8). From this result this study found that the AtMP2 should better activity compared to AtMP1.

The current result is consistent to the findings reported by E-kobon and co-workers who found the cytotoxicity of the crude *A.* *fulica* mucus against the breast cancer cell line, MCF7^46,47^. Chen et al. also found that antimicrobial peptide TH2-3 extracted from tilapia skin mucus has an effect against human fibro sarcoma cells (HT1080)^19,38^. Previous studies also suggested that the fish mucus are rich sources of antimicrobial peptides, where these peptides have a significant effect as antibacterial, antifungal, and even antiproliferative^19,48,49^. In addition, E-kobon and co-workers found that *A.* *fulica* mucus affected normal Vero cells. Importantly, the two peptides separated in the current study has no significant effect on the normal human skin fibroblast^46^.

Additionally, the current study applied Annexin V-FITC apoptosis assay to detect the apoptosis of MDA-MB-231 and MCF7 cell-lines treated with AtMP1 and AtMP2. Both AMPs) showed the capability to induce the apoptotic cancer cell death in the early and late phase at 48 h. This result is consistent with an empirical result reported by Kuo et al.^48^, who confirmed that antimicrobial peptide (MSP-4) significantly induced apoptosis in osteosarcoma MG63 cells, through an intrinsic pathway and an extrinsic pathway. The prior evidence also investigated the effects of cancer agent on cell growth and apoptosis-related gene expression in breast cancer cells^50–52^. The current study, therefore, determines the changes in gene expression of MCF‑7 and MDA-MB-231 cells treated with the AtMP1 and AtMP2 by using the human apoptosis RT^2^ Profiler PCR Array. The gene expression analysis of the current study confirmed that the AtMP1 and AtMP2 significantly increased the expression of the 5 genes of MDA-MB-231 cell, including BAX, Caspase3, Caspase7, Caspase9, and tumour suppressors TP53. BAX considered as a proapoptotic gene. In MCF7 cell, the AtMP1 and AtMP2 didn't show a significant regulation on Caspase3, yet MCF7 led to induce the apoptosis. These genes considered as tumour suppressors with a key role in cell cycle control and tumour progression. However, AtMP1 and AtMP2 significantly down-regulated the expression of antiapoptotic gene BCL-2 of MDA-MB-231 and MCF7 cells. Similarly, Murad and co-workers proved that the algal sulphated polysaccharide extract (ASPE) induces G1-phase arrest and apoptosis in MDA-MB-231 cells, which may serve as a potential therapeutic agent for breast cancer^51^. Another experimental result also found that the MCF7 cells are caspase-3 deficient with a partial deletion in the CASP-3 gene; these cells underwent cell death that lacked typical apoptotic properties^53^. However, Nan et al. confirmed that caspase-3 serves a critical function in MCF7 cell, and they suggested that caspase-3 deficiency may contribute to the chemotherapy-resistance of breast cancer^38,54^.

The extracted peptides of *A.* *testudineus* induced the cell death of MCF7 and MDA-MB-231 cells via the intrinsic apoptosis pathway, where the p53 expression led to the up-regulation of the pro-apoptotic gene BAX and downregulation of the anti-apoptotic gene BCL-2. Leading to activate the executioner caspases (caspase-9, caspase-3, caspase-7, and caspase-8) and induced apoptosis. The proteins and peptides docking databases also proved the peptide-protein interaction. The result of proteins immunoprecipitation and the LC–MS analysis proves the validity of the findings of the proteins-peptides interaction prediction and RT^2^ PCR analysis, which confirmed that AtMP1 and AtMP2 interact with tumour suppressor gene p53 and modulate the apoptosis-related proteins (caspase-9, caspase-3, caspase-7, caspase-8, p53, BCL2, and Bax), leading to induction of apoptosis and arrest the growth of breast cancer cell lines in G0/1 phase. These results were consistent with the findings reported by^42,55,56^, which found that novel web servers, such as ZDOCK and HPEPDOCK, were useful for specific blind peptide-protein docking. Zhou et al. also confirmed that the HPEPDOCK could significantly predict the native global and local protein-peptide docking compared to other servers^42,57^. This finding is in agreement with the previous studies stated that the antimicrobial peptide could inhibit the cancer cell line, such as Jurkat cells SCC-4 cell and MCF7, by modulating the expression of tumour suppressor gene p53 leading to activation of pro-apoptotic gene Bax and inhibiting the anti-apoptotic gene BCL2^56,58–61^. The result of proteins immunoprecipitation of the current study proves the validity of the findings of the proteins predictions analysis; which confirmed that AtMP1 and AtMP2 interact with tumour suppressor gene p53 and modulate the apoptosis-related proteins (caspase-3, 7, 8, 9, p53, BCL2, and Bax), leading to induction of apoptosis and arrest the growth of breast cancer cell lines in G0/1 phase.

In conclusion, the results described here clarify the fact that *A.* *testudineus* fish antimicrobial peptide functioned to inhibit MCF7 and MDA-MB-231 cells viability by inducing apoptosis. To the best of our knowledge, the present study is the first to reveal the exact mechanism of antimicrobial peptide extracted from *A.* *testudineus* fish mucus in the induction of MCF7 and MDA-MB-231 cell apoptosis. Therefore, the skin mucus of *A.* *testudineus* fish could be a notable source of a healing medicine interest toward human pathogens. This study provides a useful source for future research of cancer cell death and the change in genes that led to apoptosis, particularly in the MCF7 and MDA-MB-231 cell lines. However, the current study has tested the antimicrobial effect of *A.* *testudineus* peptides on two cancer cell line; further studies are required to examine the activity of antimicrobial peptides on several human cell lines and further characterization for these peptides’ properties.

## Supplementary Information

Supplementary Figures.

Supplementary Tables.

## Abbreviations

AMP: Antimicrobial peptides
AtMP1: First antimicrobial peptide
AtMP2: Second antimicrobial peptide
*A.**testudineus*: *Anabas* *testudineus*
ATCC: American Type Culture Collection
ATM: Ataxia-telangiectasia mutated
ATR: Ataxia-telangiectasia and Rad3 related
cDNA: Complementary deoxyribonucleic acid
CFU: Colony-forming unit
Chk1: Checkpoint 1
Chk2: Checkpoint 2
dH_2_O: Distilled water
DMEM: Dulbecco's modified eagle medium
DNA: Deoxyribonucleic acid
DSB: Double strand break
EDTA: Ethylenediaminetetraacetic acid
F2: Fraction 2
FACS: Fluorescence-activated cell sorting
FBS: Fetal bovine serum
gDNA: Genomic DNA
Gy: Gray, irradiation unit
HS27: Human foreskin fibroblast
LCMS: Liquid chromatography mass spectrometry
MCF7: Metastatic breast cancer (adenocarcinoma)
MDA-MB-231: Epithelial human breast cancer cell
NaCl: Sodium chloride
PBS: Phosphate buffered saline
PCR: Polymerase chain reaction
PI: Propidium iodide
SDS-Page: Sodium dodecyl sulfate polyacrylamide gel electrophoresis
UV: Ultraviolet
