# Supplementary material for: Anti-breast cancer synthetic peptides derived from the Anabastestudineus skin mucus fractions
Source: Sci Rep. 2021 Nov 30;11:23182. doi: 10.1038/s41598-021-02007-6 (PMC8632885; doi:10.1038/s41598-021-02007-6)
Supplement: Supplementary file 1 — Supplementary Figures. [file 41598_2021_2007_MOESM1_ESM.docx]

**Anti-breast cancer synthetic peptides derived from the *Anabas testudineus* skin mucus fractions**

Ahmed Abdul Kareem Najm^1^, Ahmad Azfaralariff^2,4^_,_ Herryawan Ryadi Eziwar Dyari^3^, Babul Airianah Othman^2^, Muhammad Shahid^2^, Nahid Khalili^1^, Douglas Law^5^, Sharifah Sakinah Syed Alwi^6^ & Shazrul Fazry^2,4,7^

*^1^Department of Biological Sciences and Biotechnology, Faculty of Science and Technology, Universiti Kebangsaan Malaysia, 43600, Bangi, Selangor Darul Ehsan, Malaysia*

*^. 2^ Department of Food Sciences, Faculty of Science and Technology, Universiti Kebangsaan Malaysia, 43600, Bangi, Selangor Darul Ehsan, Malaysia.*

*^3^Department of Earth Sciences, Faculty of Science and Technology, Universiti Kebangsaan Malaysia, 43600, Bangi, Selangor Darul Ehsan, Malaysia.*

*^4^ Innovative Center for Confectionery Technology (MANIS), Faculty of Science and Technology, Universiti Kebangsaan Malaysia, 43600, Bangi, Selangor Darul Ehsan, Malaysia.*

*^5^Faculty of Health and Life Sciences, Inti International University, Persiaran Perdana BBN Putra Nilai, 71800 Nilai, Negeri Sembilan*

*^6^Department of Biomedical Science, Faculty of Medicine & Health Sciences, Universiti Putra Malaysia, 43400, UPM Serdang, Selangor Darul Ehsan, Malaysia.*

*^7^ Chini Lake Research Centre, Faculty of Science and Technology, Universiti Kebangsaan Malaysia, 43600, Bangi, Selangor Darul Ehsan, Malaysia.*

***Corresponding e-mail:** [***shazrul@ukm.edu.my***](mailto:shazrul@ukm.edu.my)

**Orcid id: *0000-0002-7072-8609***

**Tel: +60192725203**


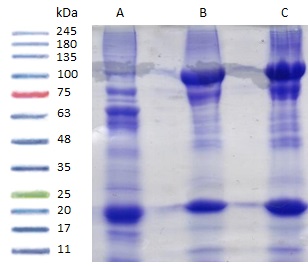


**Supplementary Figure S1.** Protein profile for crude and fraction by 12% SDS PAGE from Sephadex G-50. A represents 1^st^ replicate, B represents 2^nd^ replicate, C represents 3^rd^ replicate (related to figure3a).


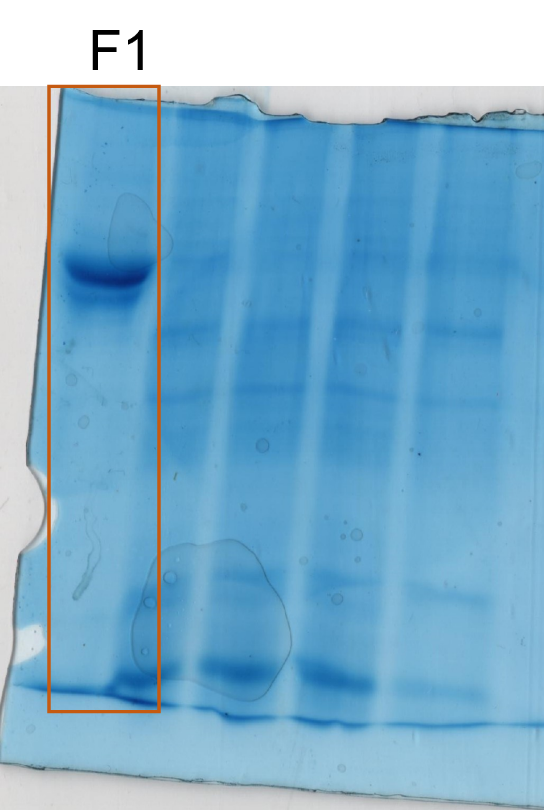


**Supplementary Figure S2.** SDS-Page of fractions (F1) (related to figure 3B).

**
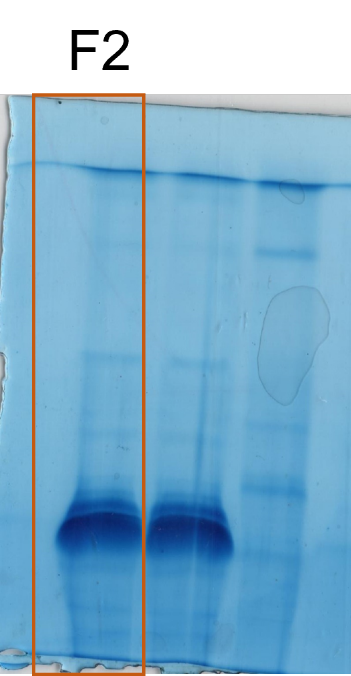
**

**Supplementary Figure S3.** SDS-Page of fractions (F2) (related to figure 3B).


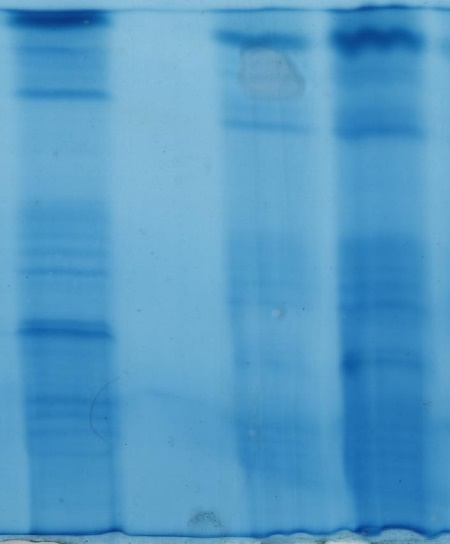


F3

**Supplementary Figure S4.** SDS-Page of fractions (F3) (related to figure 3B).


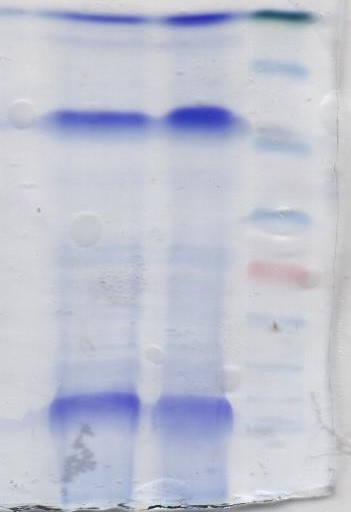


F4

**Supplementary Figure S5.** SDS-Page of fractions (F4) (related to figure 3B).

**
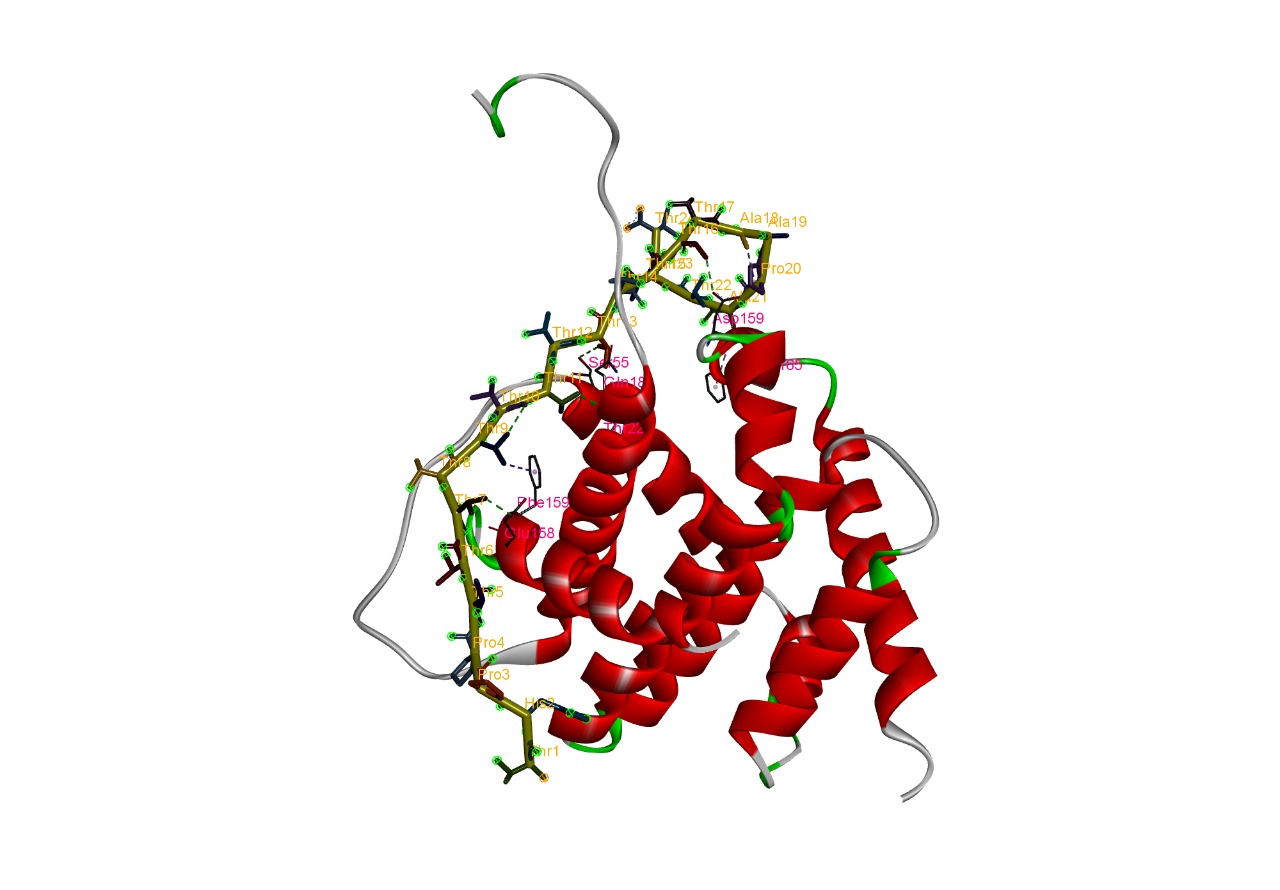
**

**Supplementary Figure S6.** Peptide-protein docking 3D model by using HPEDOCK for (A) AtMP1+BAX (related to figure 14A).

**
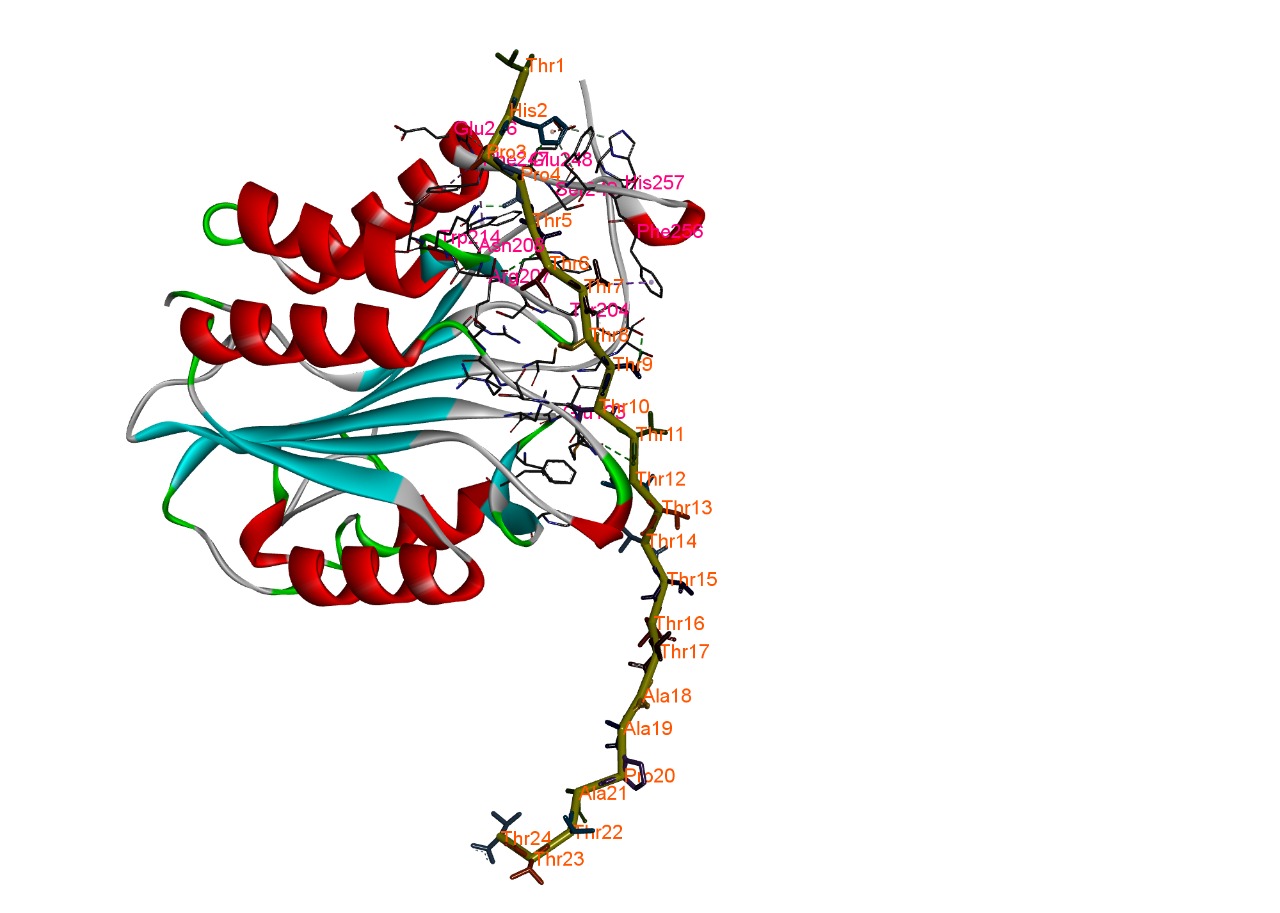
**

**Supplementary Figure S7.** Peptide-protein docking 3D model by using HPEDOCK for (B) AtMP1+Caspase3 (related to figure 14B).

**
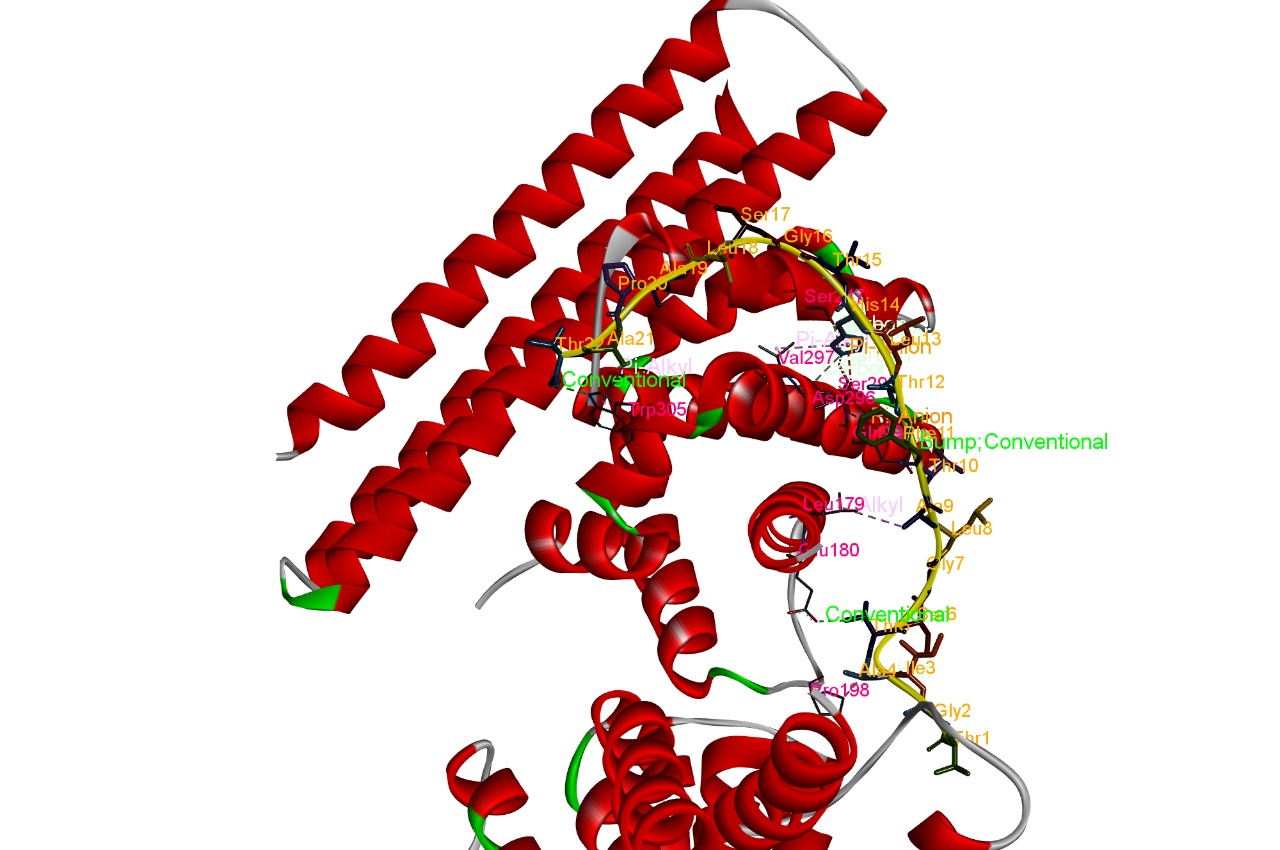
**

**Supplementary Figure S8.** Peptide-protein docking 3D model by using HPEDOCK for (C) AtMP1+Caspase9 (related to figure 14C).

**
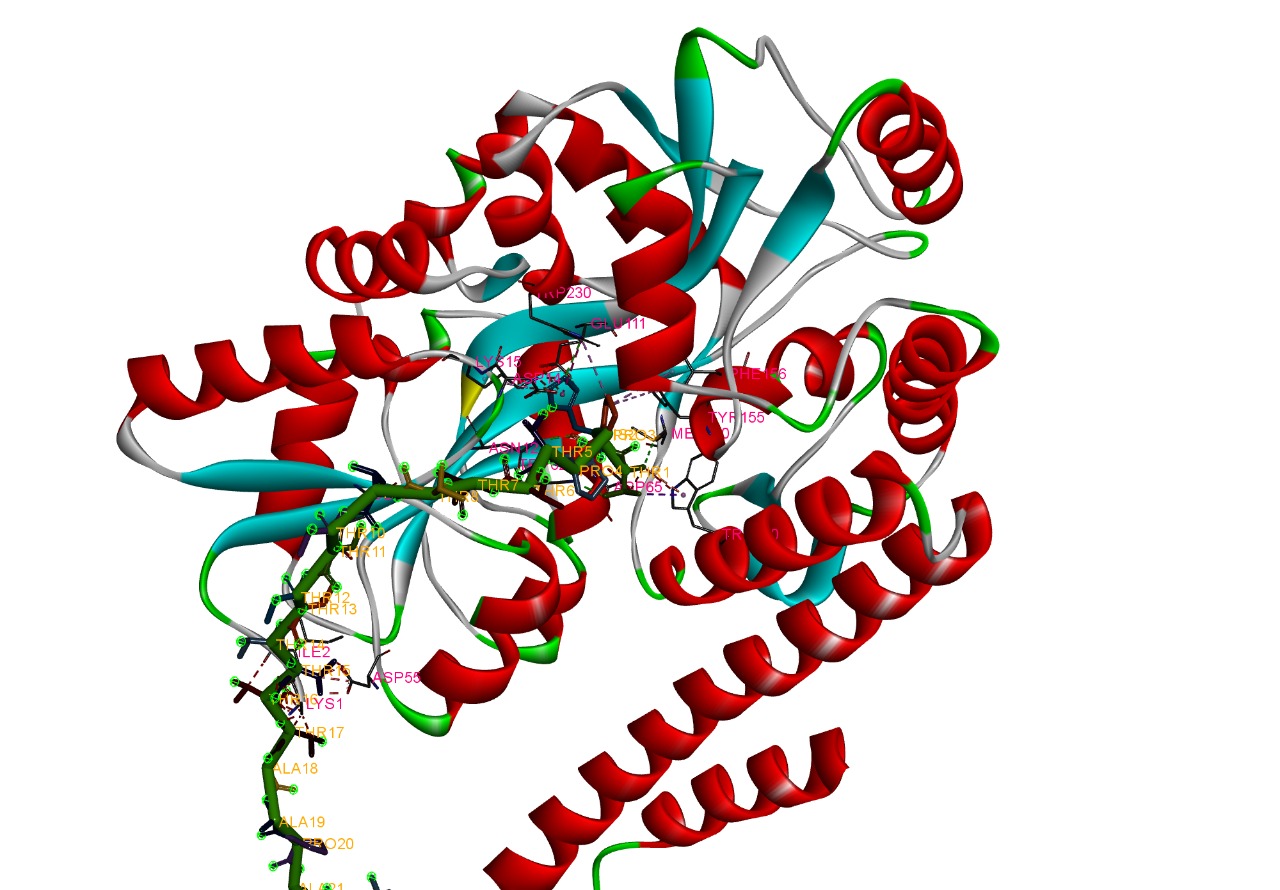
**

**Supplementary Figure S9.** Peptide-protein docking 3D model by using HPEDOCK for (D) AtMP1+P53 (related to figure 14D).

**
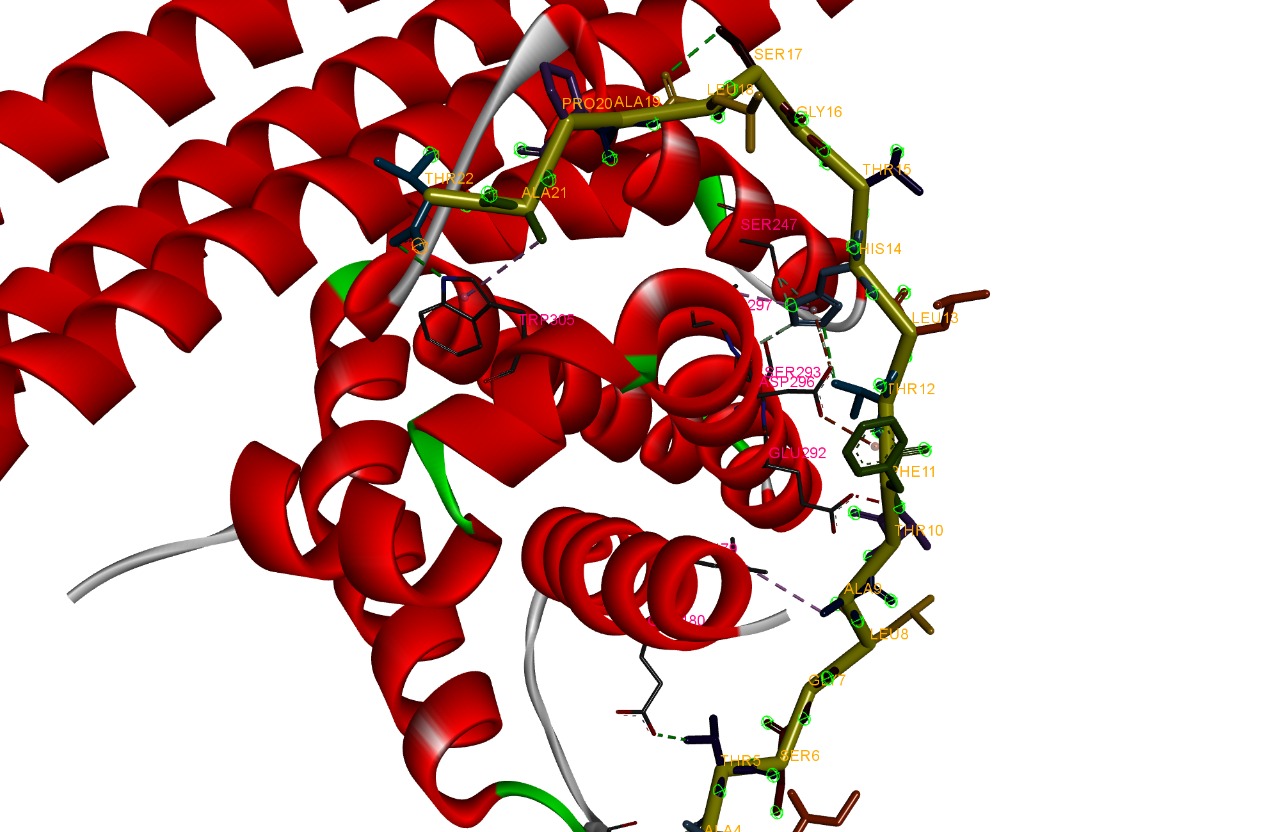
**

**Supplementary Figure S10.** Peptide-protein docking 3D model by using HPEDOCK for (E) AtMP1+BCL2 (related to figure 14E).


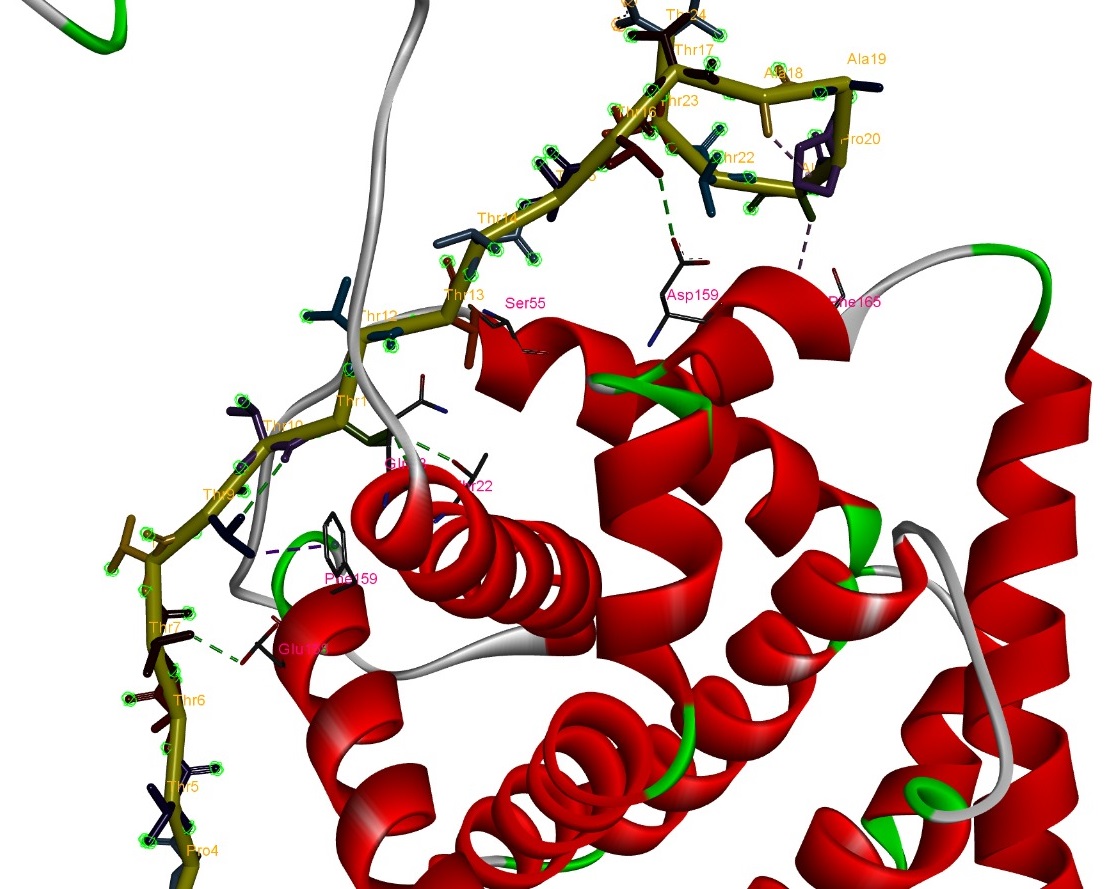


**Supplementary Figure S11.** Peptide-protein docking 3D model by using HPEDOCK for (F) AtMP2+BAX (related to figure 14F).


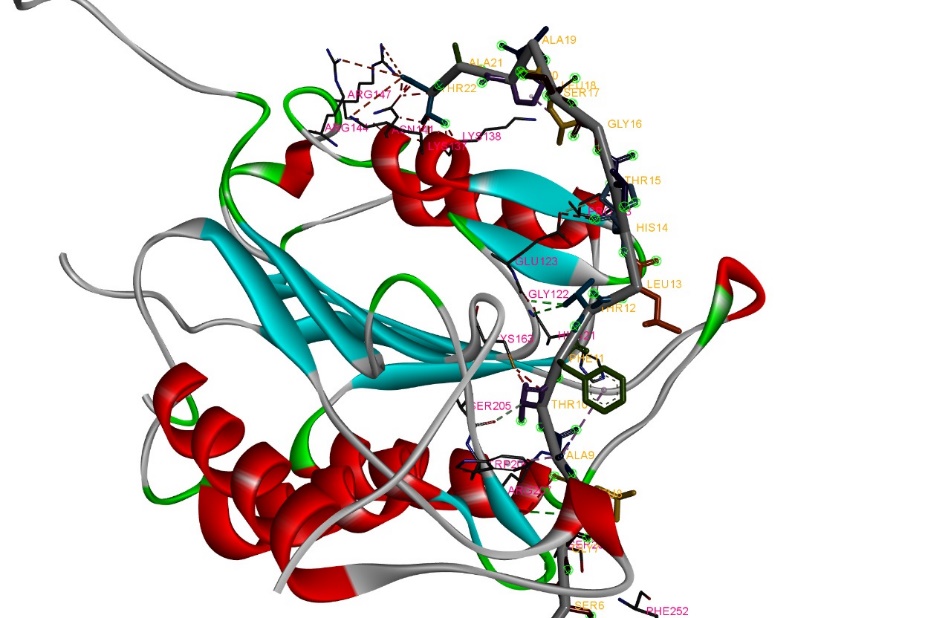


**Supplementary Figure S12.** Peptide-protein docking 3D model by using HPEDOCK for (G) AtMP2+ caspase3 (related to figure 14G).


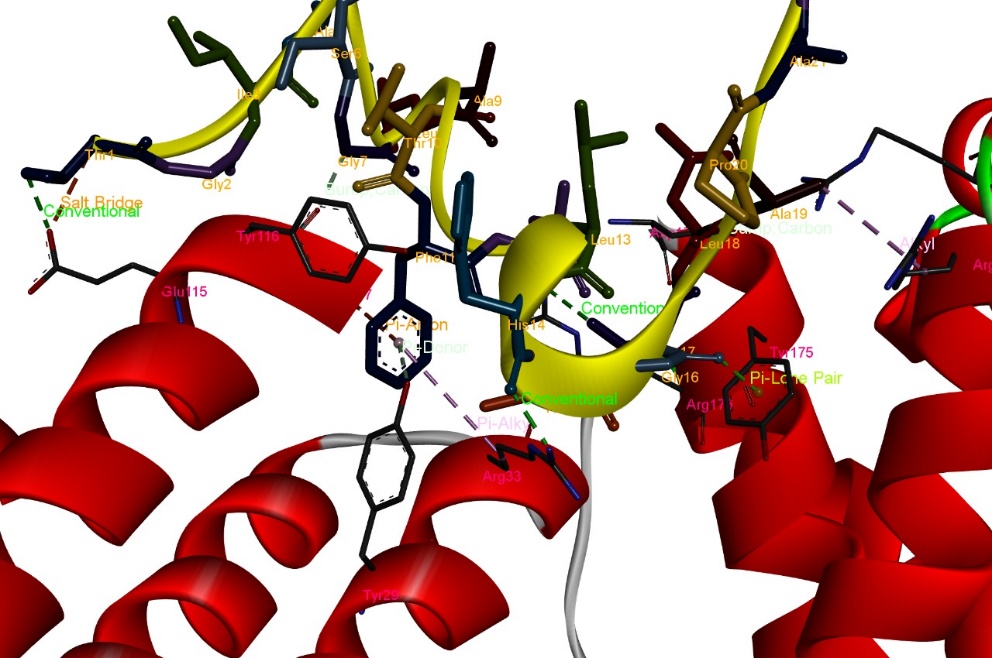


**Supplementary Figure S13.** Peptide-protein docking 3D model by using HPEDOCK for (H) AtMP2+ Caspae7 (related to figure 14H).


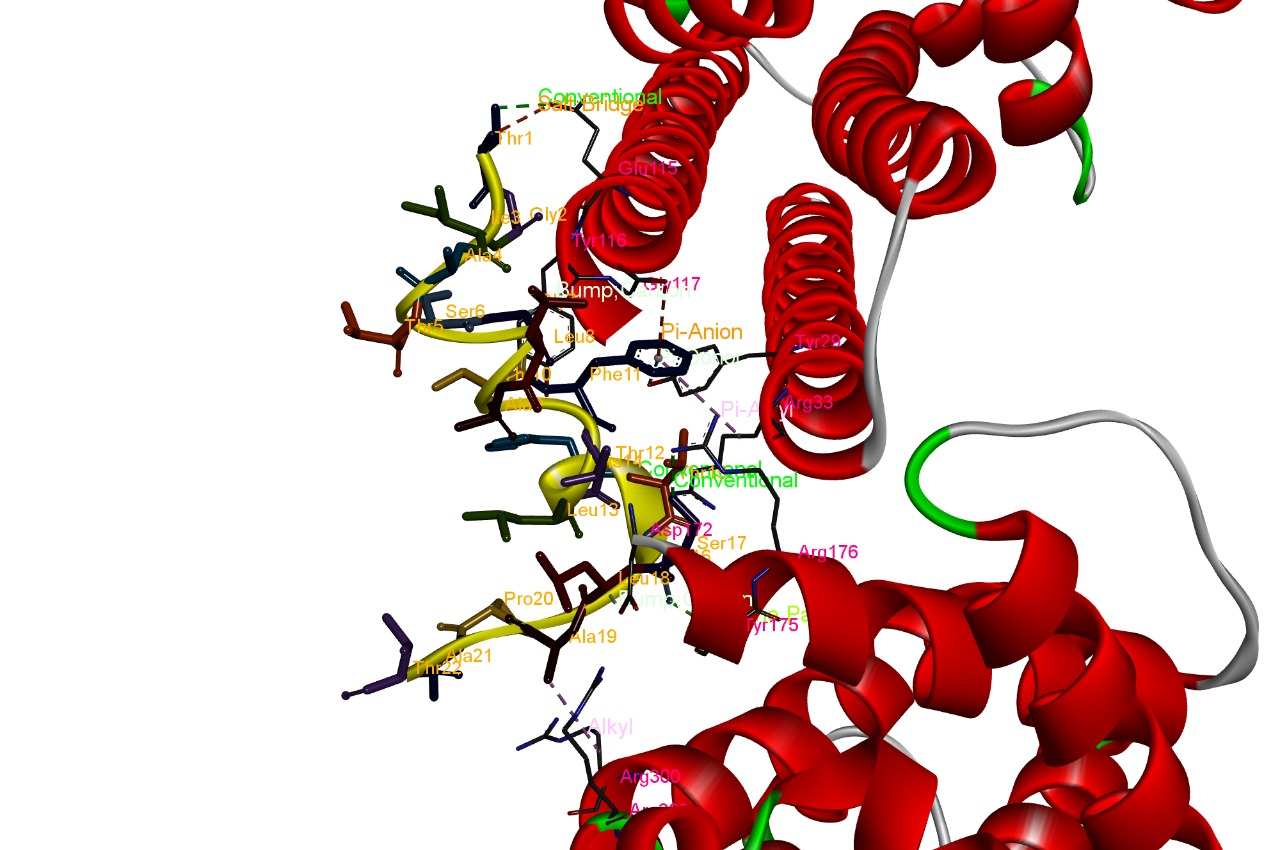


**Supplementary Figure S14.** Peptide-protein docking 3D model by using HPEDOCK for (I) AtMP2+ Caspase8 (related to figure 14I).


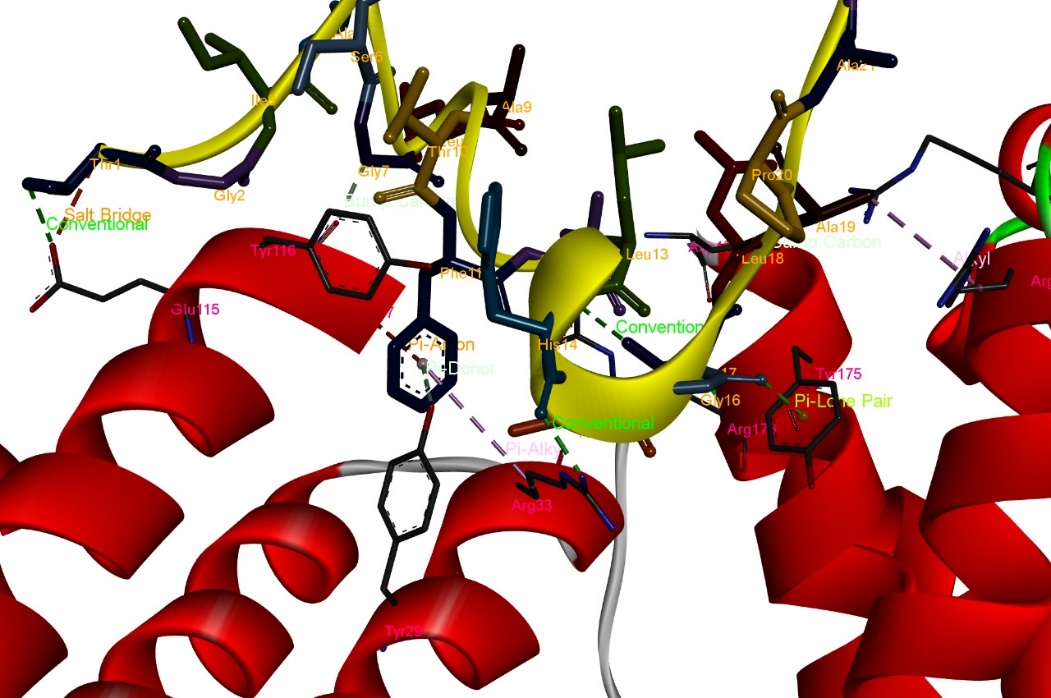


**Supplementary Figure S15.** Peptide-protein docking 3D model by using HPEDOCK for (J)

AtMP2+ Caspase9 (related to figure 14J).


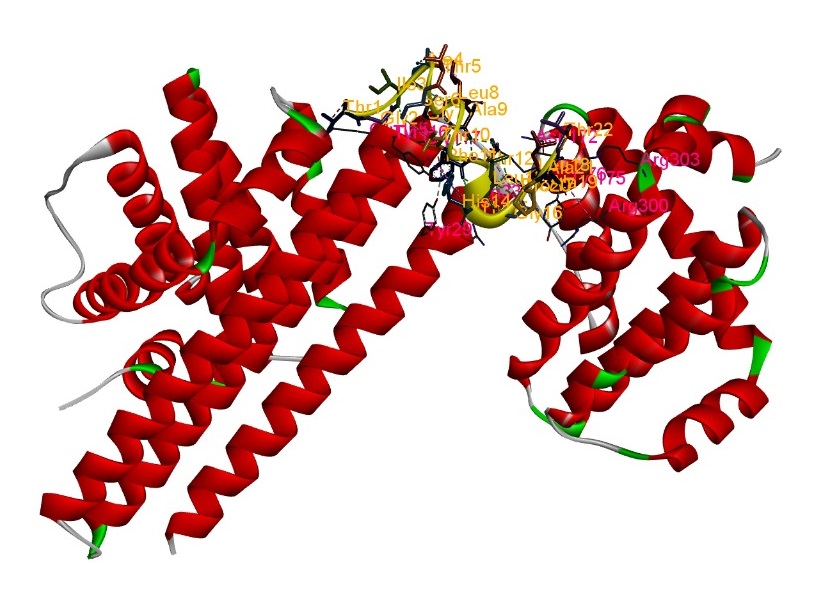


**Supplementary Figure S16.** Peptide-protein docking 3D model by using HPEDOCK for (K)

AtMP2+ P53 (related to figure 14K).


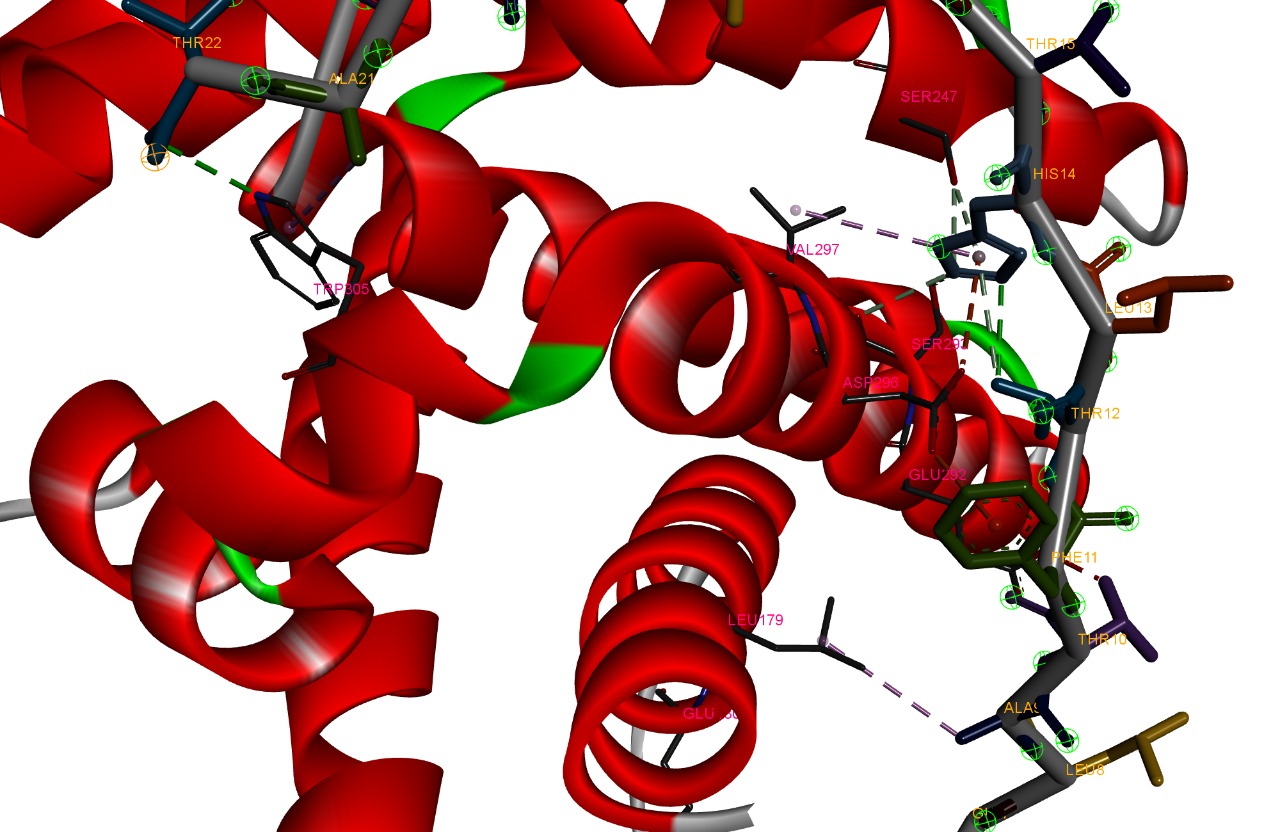


**Supplementary Figure S17.** Peptide-protein docking 3D model by using HPEDOCK for (L)

AtMP2+ BCL2 (related to figure 14L).

**
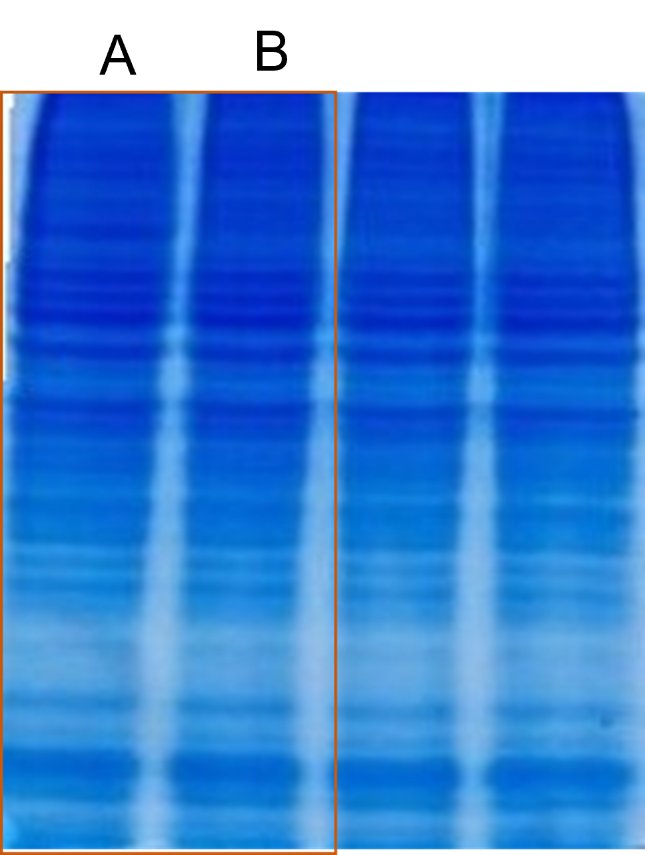
**

**Supplementary Figure S18.** Total protein cell lysate before pulldown (related to figure 16A).


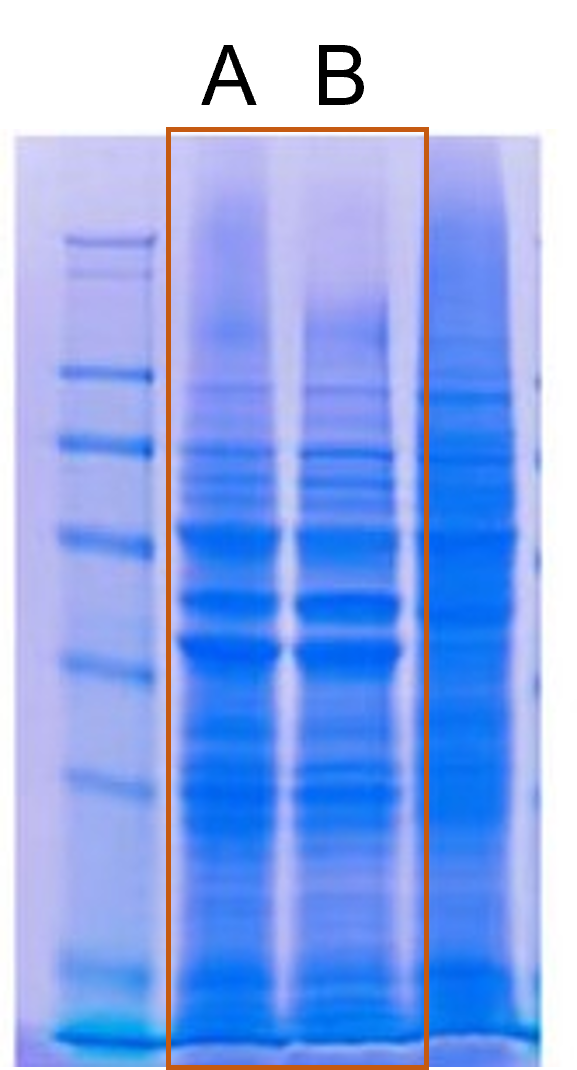


**Supplementary Figure S19.** Mitochondrial protein cell lysate before pulldown (related to figure 16A).

**
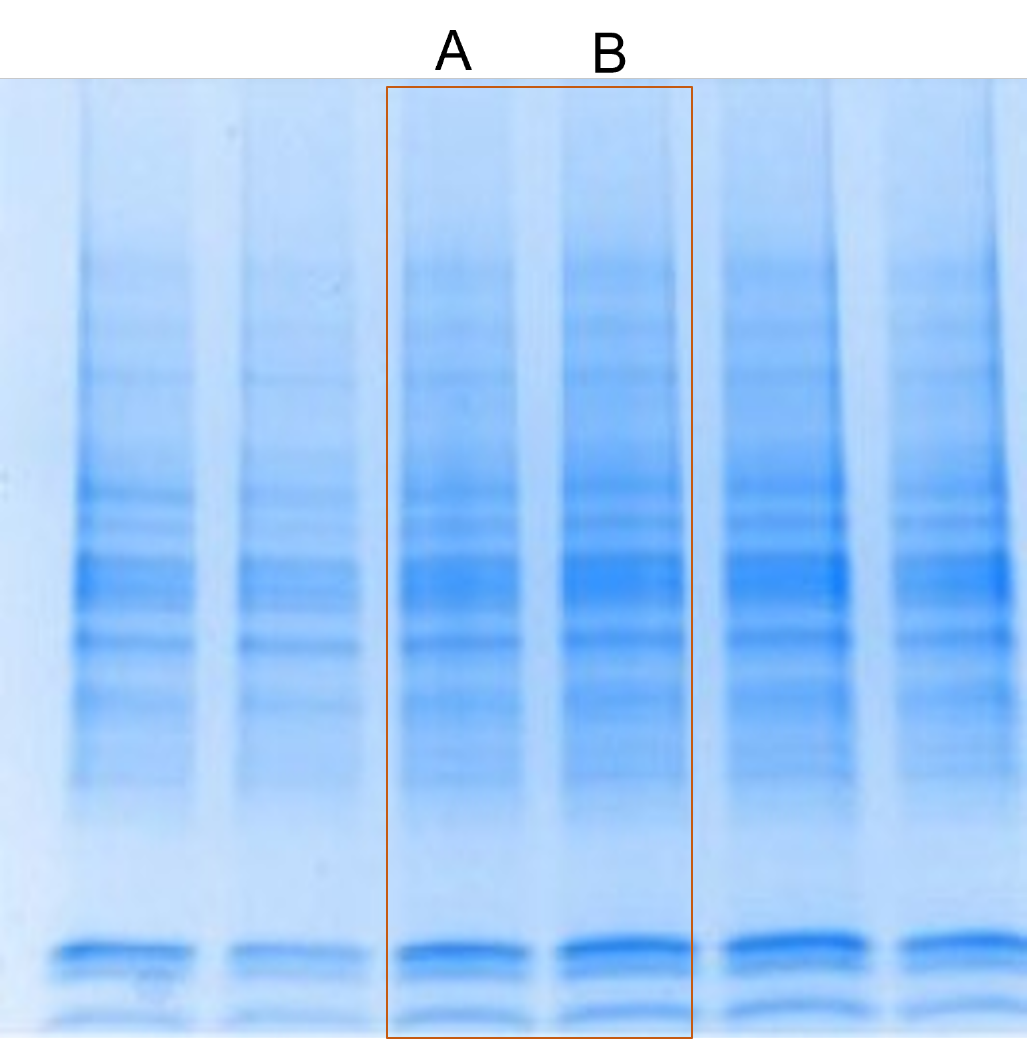
**

**Supplementary Figure S20.** Nucleus protein cell lysate before pulldown (related to figure 16A).

**
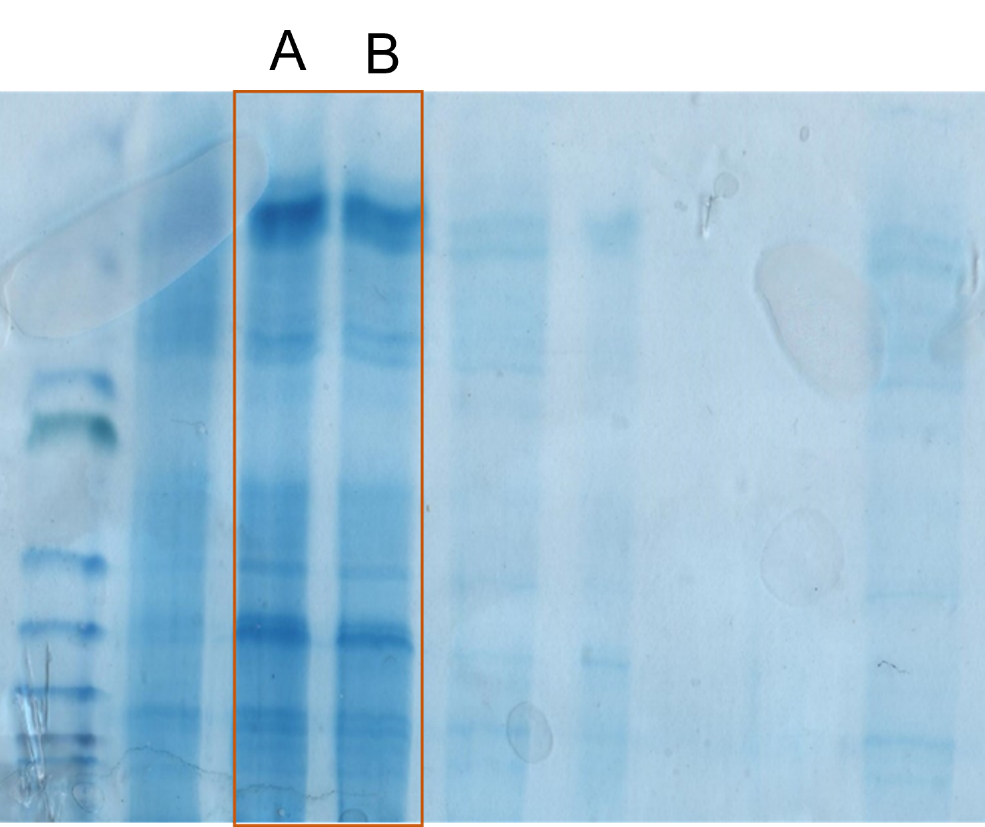
**

**Supplementary Figure S21 .** Membrane proteins cell lysate before pulldown (related to figure 16A).


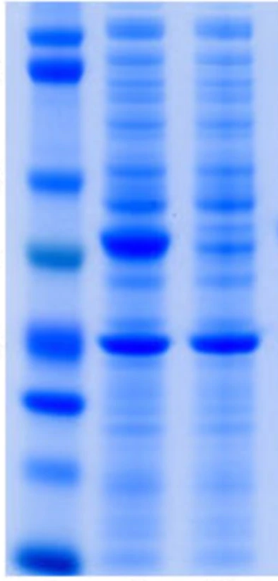


A

B

Caspase 3

Caspase 7

Caspase 8

BCL2

BAX

Caspase 9

P53

**Supplementary Figure S22.** Total proteins cell lysate after pulldown ***(BA)*** total proteins linked to AtMP1, ***(BB)*** total proteins linked to AtMP2 (related to figure 16B).

**
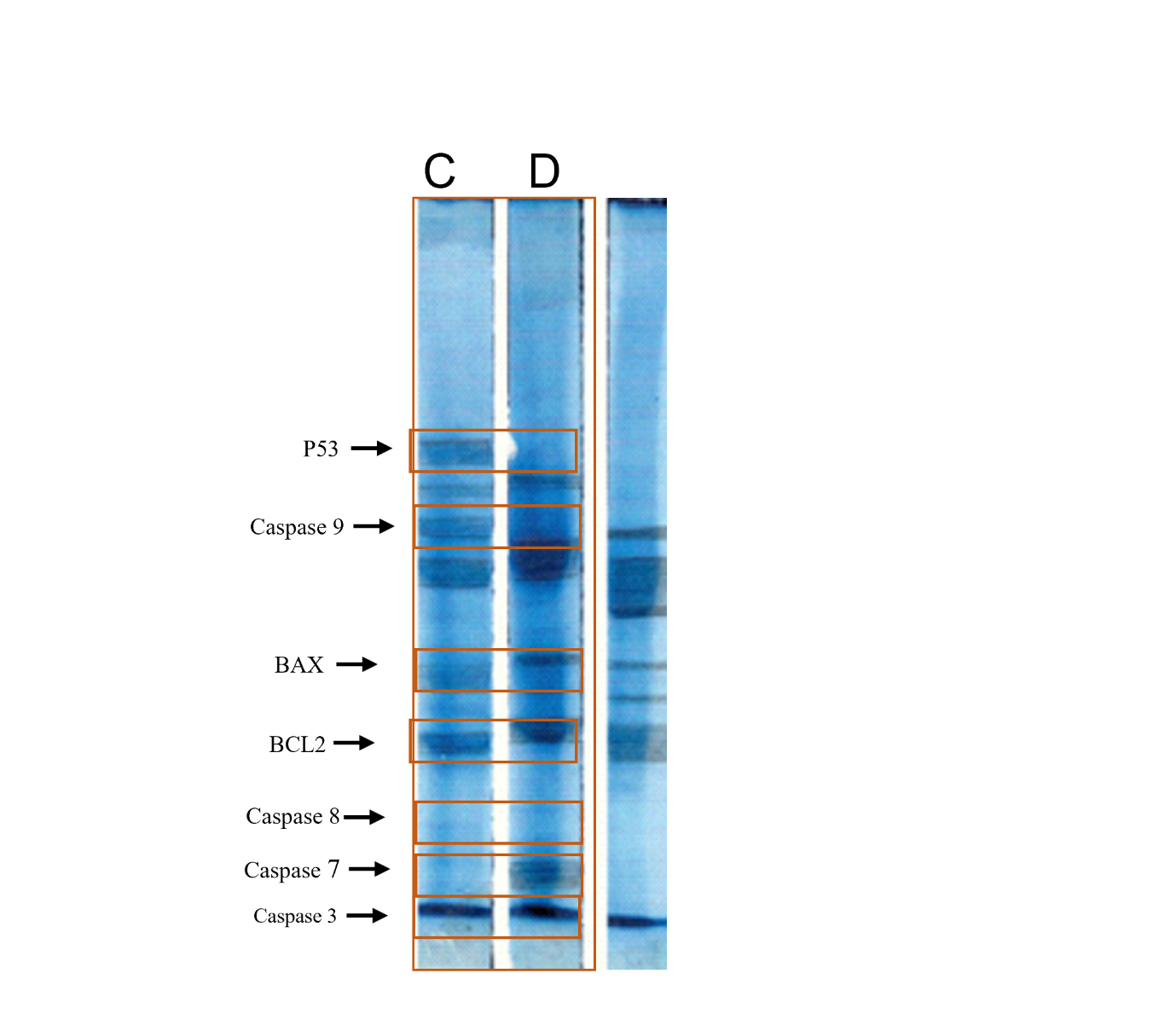
**

**Supplementary Figure S23.** Membrane protein after pulldown ***(BC)*** membrane proteins linked to AtMP1, and ***(BD)*** membrane proteins linked to AtMP2 (related to figure 16B).

**
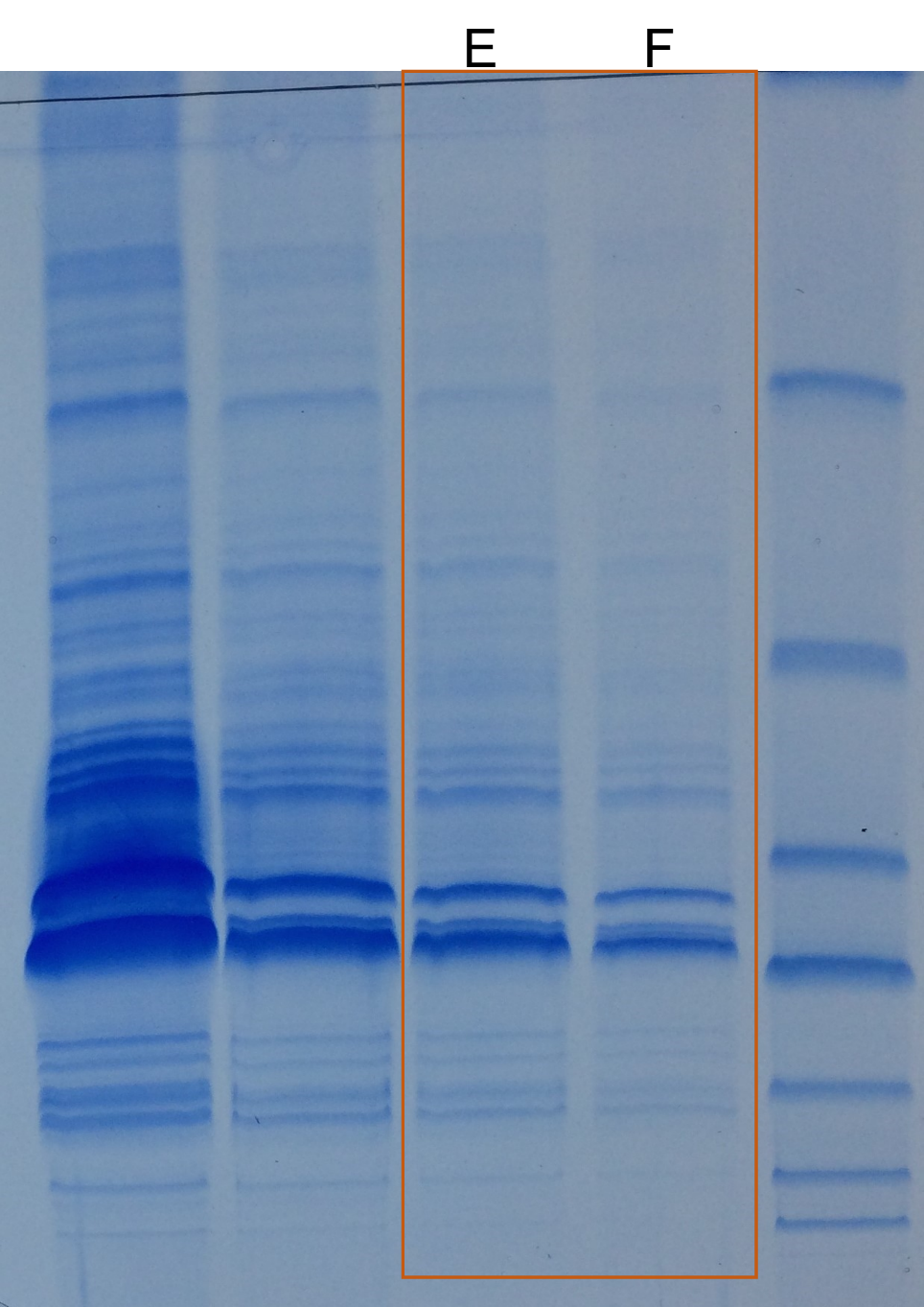
**

**Supplementary Figure S24.** Mitochndrial protein cell lysate after pulldown *(****BE****)* mitochondrial proteins linked to AtMP1, and *(****BF****)* mitochondrial proteins linked to AtMP2. (Related to figure 16B).

**
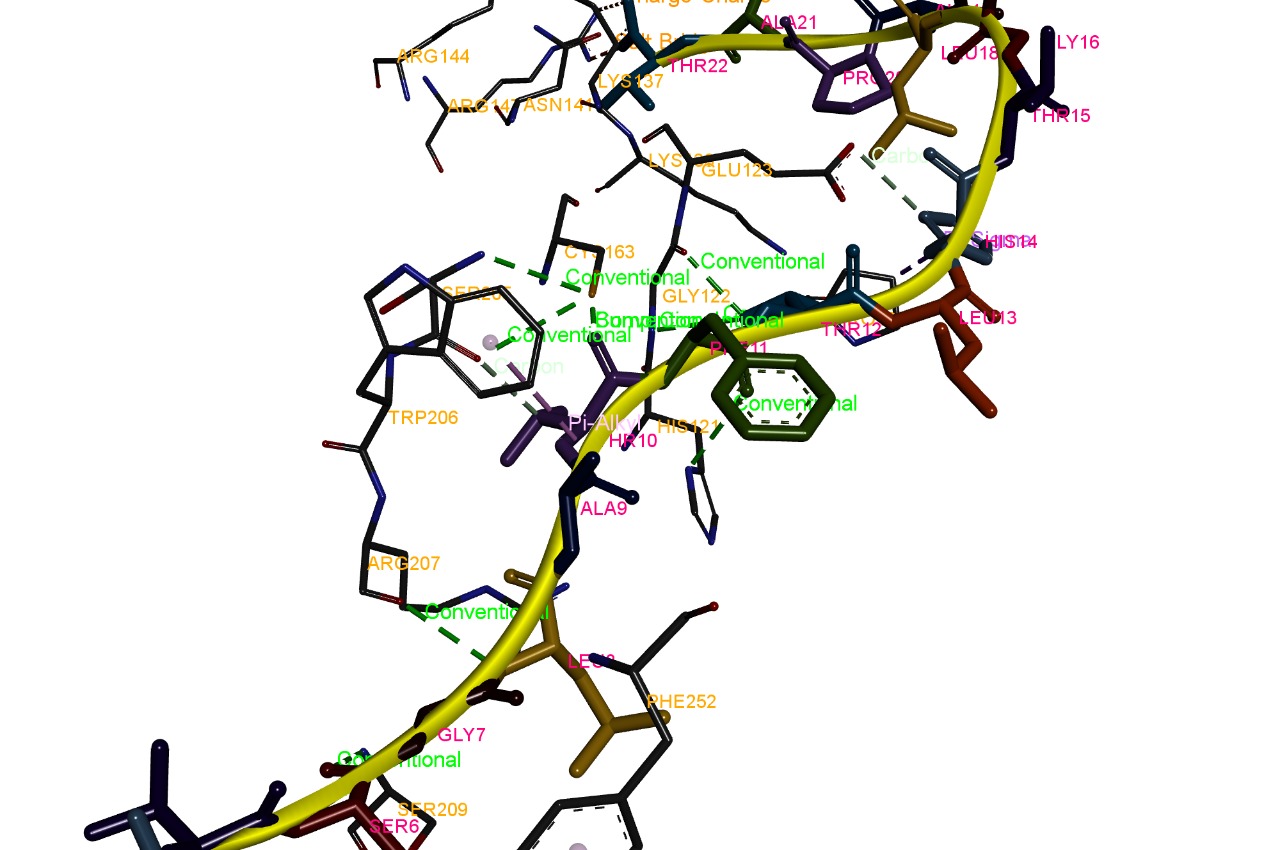
**

**Supplementary Figure S25.** Peptide-protein docking 3D model by using HPEDOCK for (A)

AtMP2+ BAX (related to figure 15A).


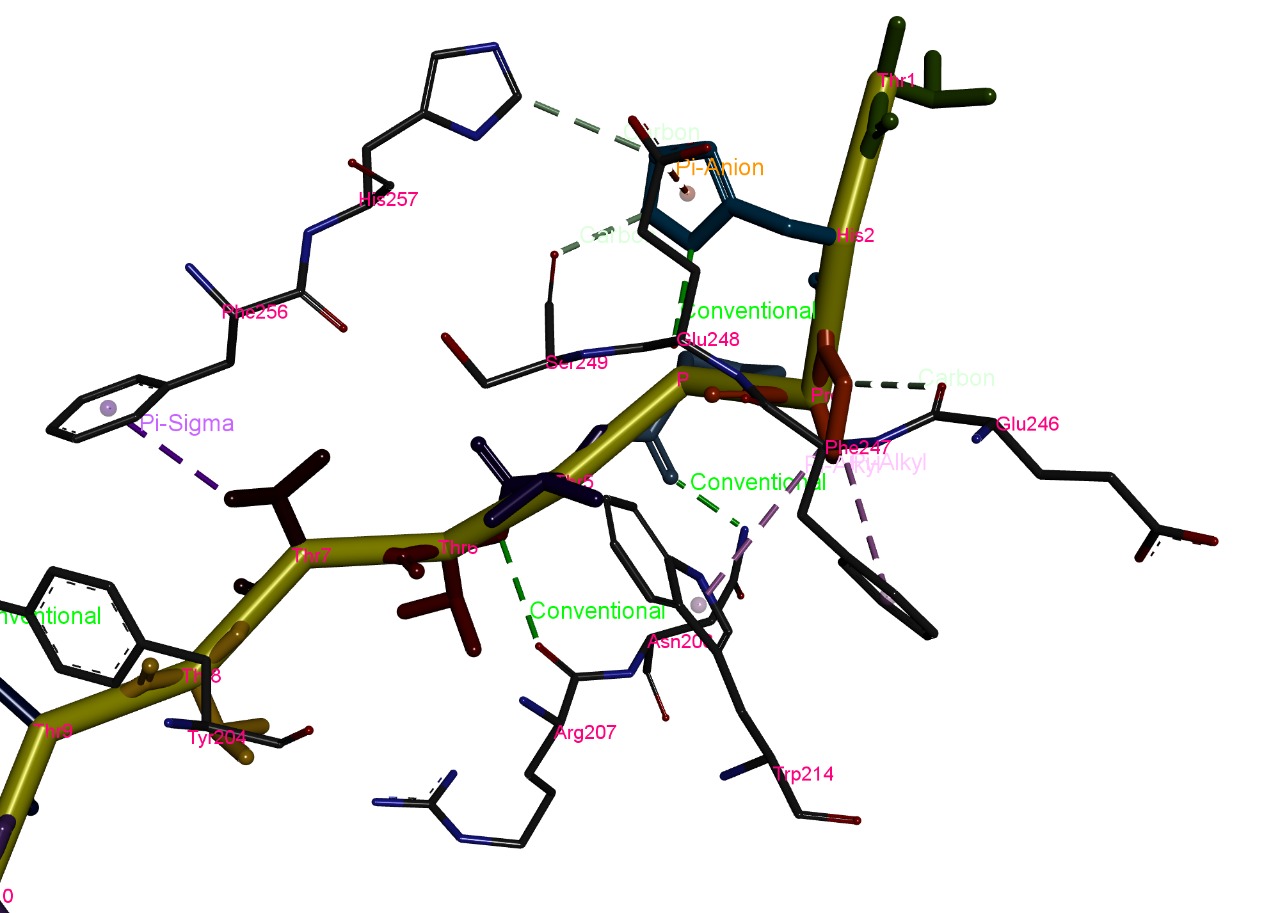


**Supplementary Figure S26.** Peptide-protein docking 3D model by using HPEDOCK for (B)

AtMP2+ Caspase 3 (related to figure 15B).


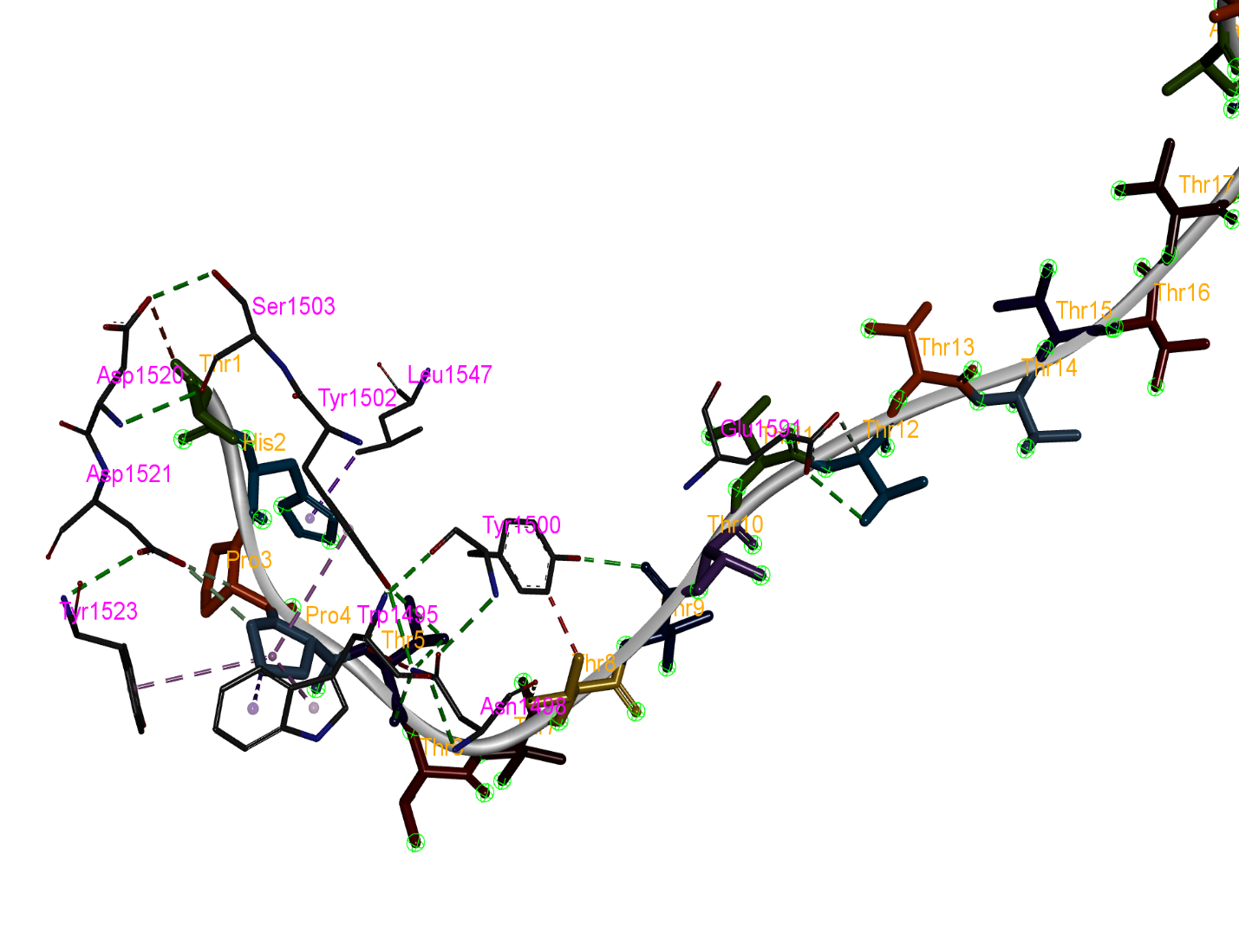


**Supplementary Figure S27.** Peptide-protein docking 3D model by using HPEDOCK for (C)

AtMP2+ Caspase 7 (related to figure 15C).


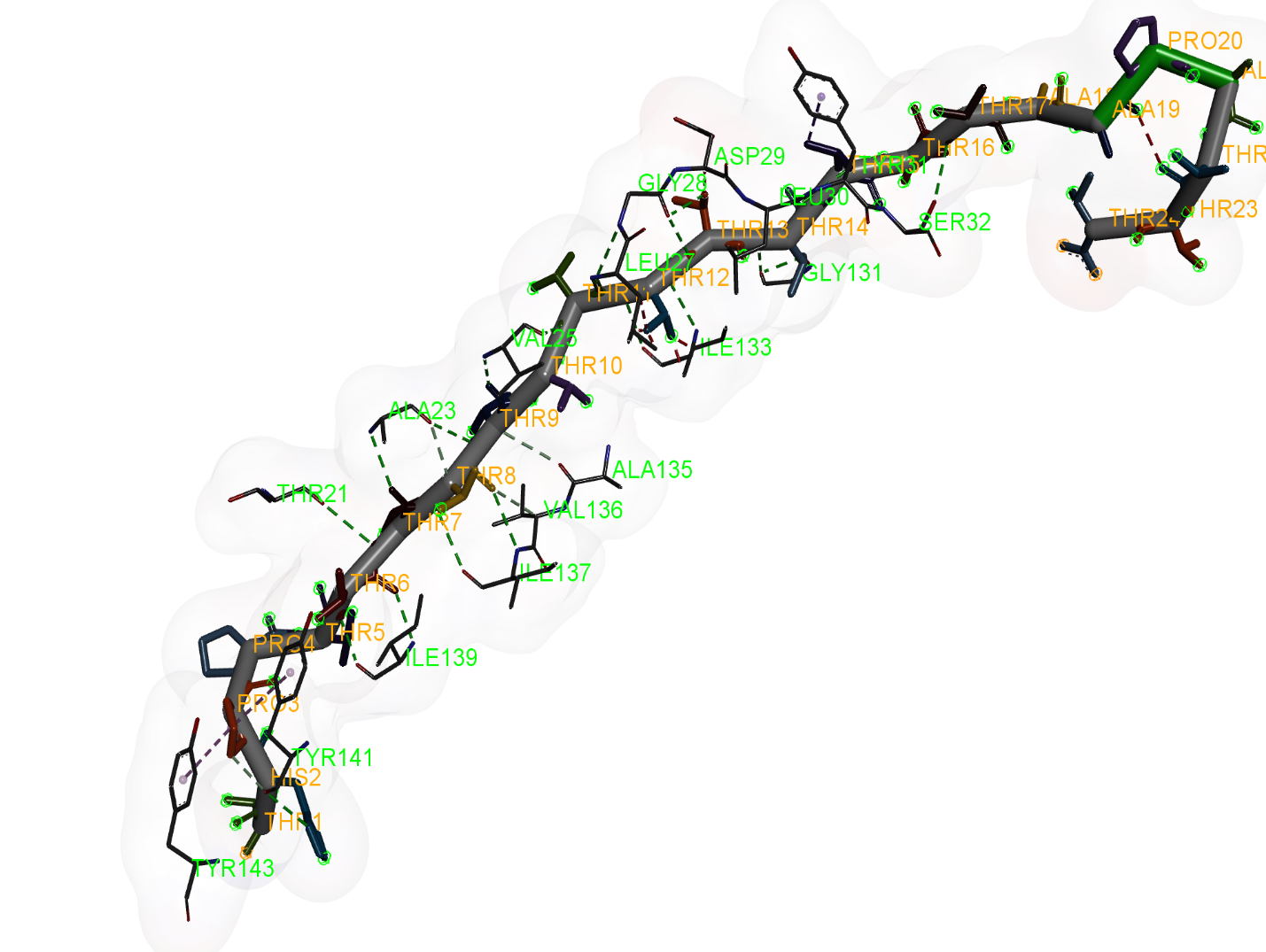


**Supplementary Figure S28.** Peptide-protein docking 3D model by using HPEDOCK for (D)

AtMP2+ Caspase 8 (related to figure 15D).


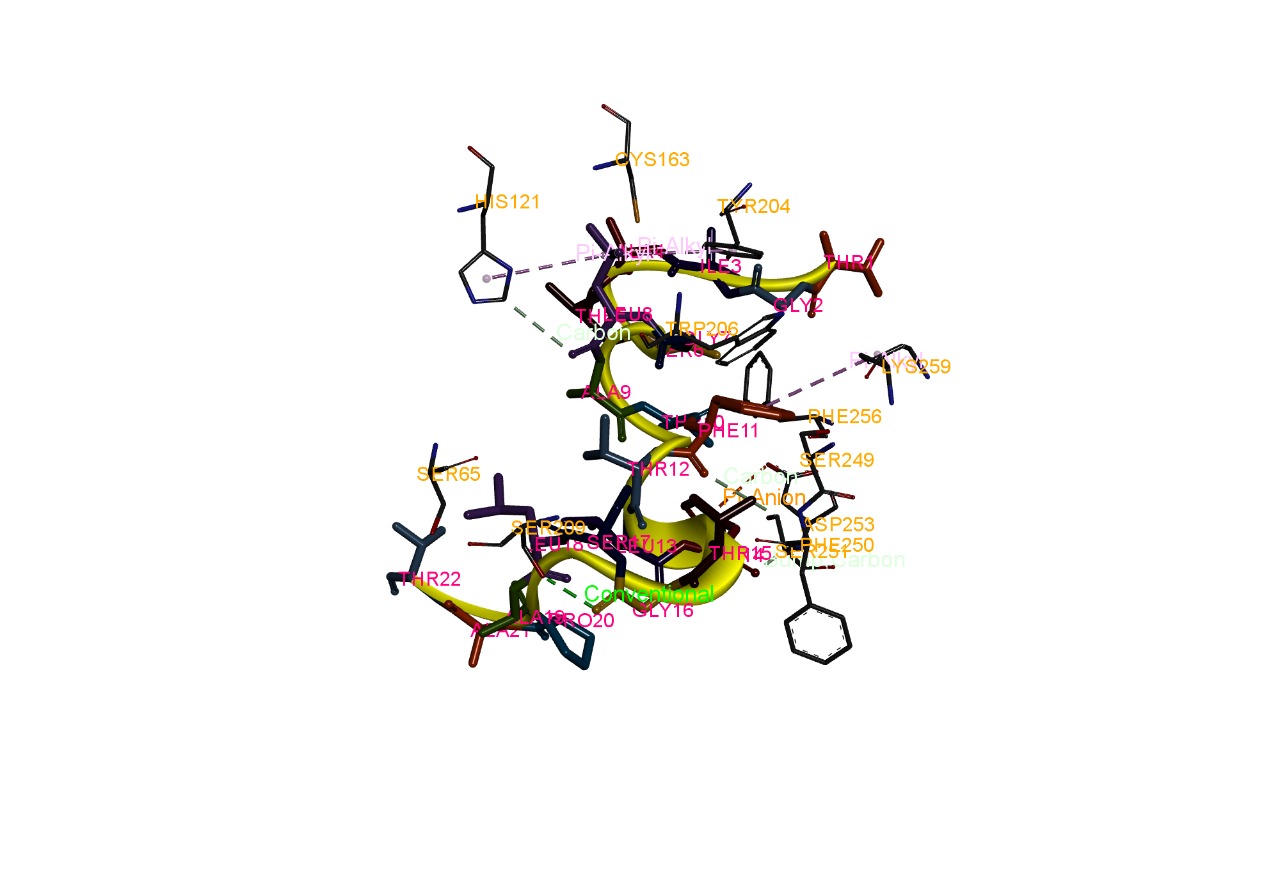


**Supplementary Figure S29.** Peptide-protein docking 3D model by using HPEDOCK for (E)

AtMP2+ Caspase 9 (related to figure 15E).


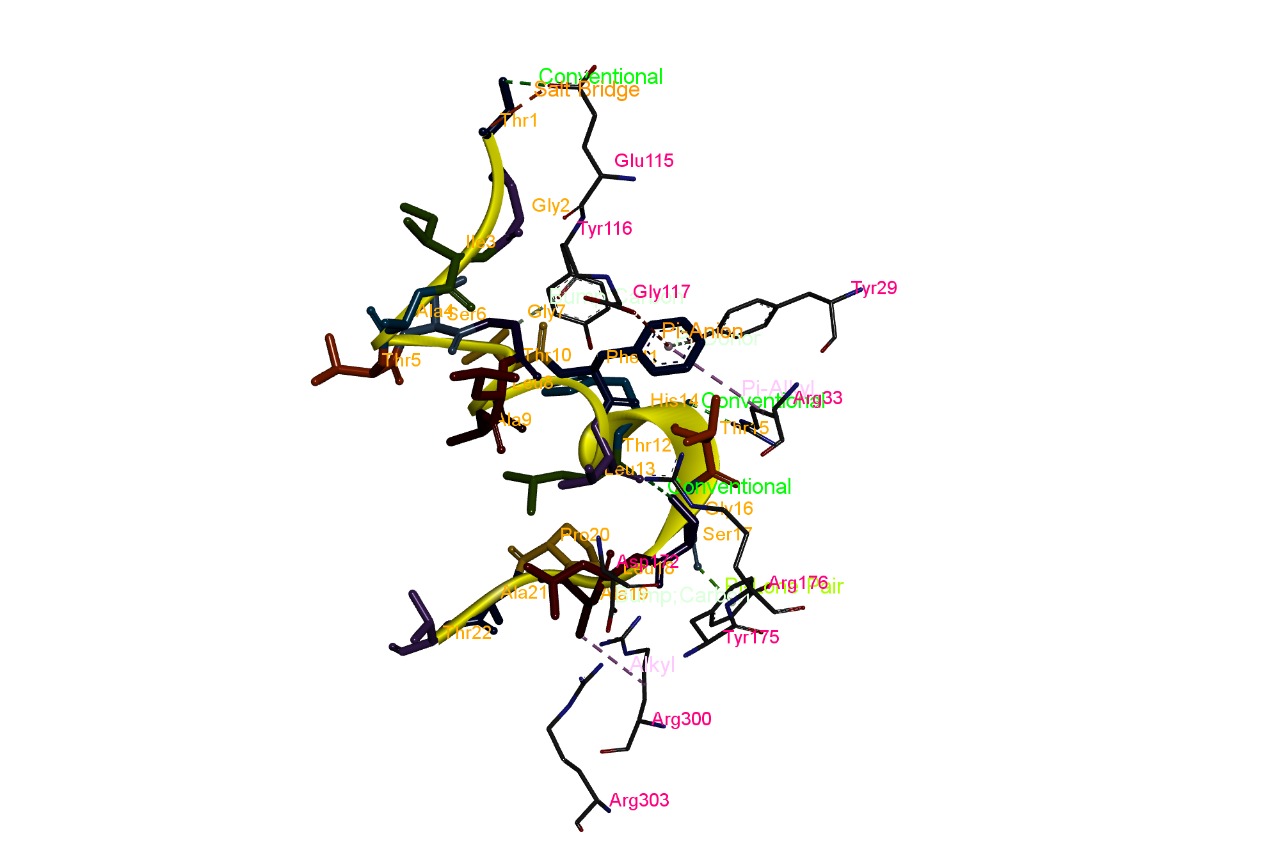


**Supplementary Figure S30.** Peptide-protein docking 3D model by using HPEDOCK for (F)

AtMP2+ P53 (related to figure 15E).


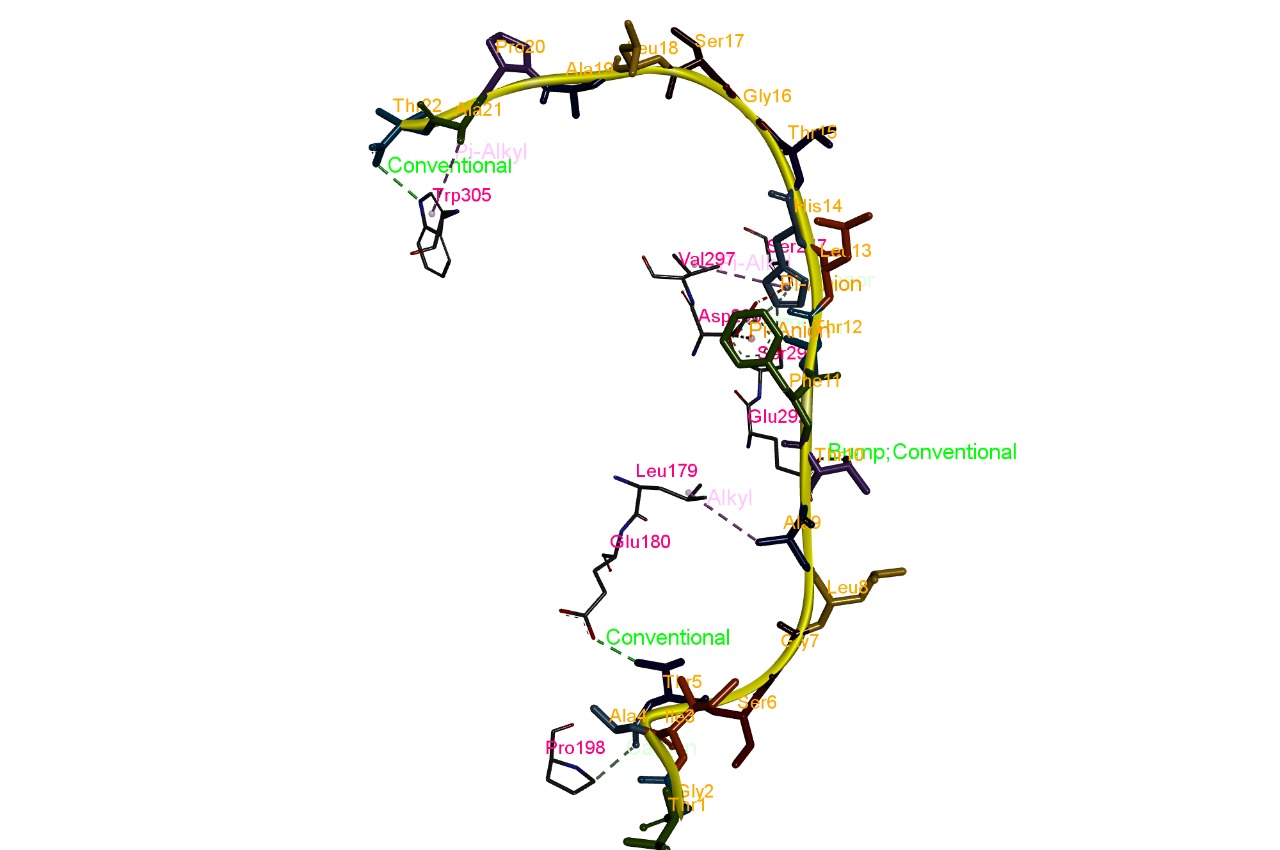


**Supplementary Figure S31.** Peptide-protein docking 3D model by using HPEDOCK for (G)

AtMP2+ BCL2 (related to figure 15G).
