# Supplementary material for: Anti-breast cancer synthetic peptides derived from the Anabastestudineus skin mucus fractions
Source: Sci Rep. 2021 Nov 30;11:23182. doi: 10.1038/s41598-021-02007-6 (PMC8632885; doi:10.1038/s41598-021-02007-6)
Supplement: Supplementary file 2 — Supplementary Tables. [file 41598_2021_2007_MOESM2_ESM.pdf]

Anti-breast cancer synthetic peptides derived from the *Anabas testudineus* skin mucus fractions

Ahmed Abdul Kareem Najm[1], Ahmad Azfaralariff[2,4], Herryawan Ryadi Eziwar Dyari[3], Babul Airianah Othman[2], Muhammad Shahid[1], Nahid Khalili[1], Douglas Law[5], Sharifah Sakinah Syed Alwi[6] & Shazrul Fazry[2,4,7]

[1]*Department of Biological Sciences and Biotechnology, Faculty of Science and Technology, Universiti Kebangsaan Malaysia, 43600, Bangi, Selangor Darul Ehsan, Malaysia*

[2] *Department of Food Sciences, Faculty of Science and Technology, Universiti Kebangsaan Malaysia, 43600, Bangi, Selangor Darul Ehsan, Malaysia.*

[3]*Department of Earth Sciences, Faculty of Science and Technology, Universiti Kebangsaan Malaysia, 43600, Bangi, Selangor Darul Ehsan, Malaysia.*

[4] *Innovative Center for Confectionery Technology (MANIS), Faculty of Science and Technology, Universiti Kebangsaan Malaysia, 43600, Bangi, Selangor Darul Ehsan, Malaysia.*

[5]*Faculty of Health and Life Sciences, Inti International University, Persiaran Perdana BBN Putra Nilai, 71800 Nilai, Negeri Sembilan*

[6]*Department of Biomedical Science, Faculty of Medicine & Health Sciences, Universiti Putra Malaysia, 43400, UPM Serdang, Selangor Darul Ehsan, Malaysia.*

[7] *Chini Lake Research Centre, Faculty of Science and Technology, Universiti Kebangsaan Malaysia, 43600, Bangi, Selangor Darul Ehsan, Malaysia.*

Corresponding e-mail:** ***shazrul@ukm.edu.my

Orcid id: ***0000-0002-7072-8609***

Tel: +60192725203

Supplementary Table S1. the gene list of PCR Array for MDA-MB-231 cancer cells treated with the (AtMP1) (related to figure 11C).

Position	Gene Symbol	Fold Regulation	p-Value	comment
A09	BAX	3.36	nan	
C06	CASP14	2.61	nan	
C08	CASP3	4.26	nan	
C09	CASP4	3.63	nan	
C12	CASP7	3.28	nan	
D02	CASP9	3.36	nan	
F05	PYCARD	2.61	nan	
G06	TP53	3.12	nan	
H05	RPLP0	3.37	nan	
A11	BCL2	-2.01	nan	

Supplementary Table S2. the gene list of PCR Array for MDA-MB-231 cancer cells treated with the (AtMP2) (related to figure 11D).

Position	Gene Symbol	Fold Regulation	p-Value	comment
A09	BAX	3.32	nan	
A10	BCL10	2.02	nan	
C06	CASP14	2.58	nan	
C08	CASP3	4.20	nan	
C12	CASP7	3.25	nan	
D01	CASP8	3.95	nan	
D02	CASP9	3.32	nan	
G06	TP53	3.07	nan	
H05	RPLP0	3.32	nan	
A11	BCL2	-4.06	nan	

Supplementary Table S3. the gene list of PCR Array for MCF7 cancer cells treated with the (AtMP1) (related to figure 12C).

Position	Gene Symbol	Fold Regulation	p-Value	comment
A09	BAX	2.86	0.001227	
C08	CASP3	2.55	0.018053	
C12	CASP7	2.96	0.0027	
G06	TP53	4.16	0.000011	
A11	BCL2	-6.02	0.000012	

Supplementary Table S4. the gene list of PCR Array for MCF7 cancer cells treated with the (AtMP2) (related to figure 12D).

Position	Gene Symbol	Fold Regulation	p-Value	comment
A05	BAD	3.21	0.002381	
A09	BAX	4.46	0.000004	
C08	CASP3	0.12	0.00000	
C12	CASP7	2.92	0.000001	
D01	CASP8	2.20	0.00011	
D02	CASP9	2.18	0.00171	
G06	TP53	4.83	0.000001	
A11	BCL2	-2.91	0.022057	

Supplementary Table S5. Database websites and date of access

**Data base**	**Website**	**Date of access**
ADAM	*http://bioinformatics.cs.ntou.edu.tw/ADAM*	02/07/2020
iAMP-2L	*http://www.jci-bioinfo.cn/iAMP-2L.*	02/07/2020
CAMP-R3	*http://www.camp3.bicnirrh.res.in/*	02/07/2020
AMPfun	*http://fdblab.csie.ncu.edu.tw/AMPfun/about.html*	02/07/2020
ADp3	*APD, http://aps.unmc.edu/AP/*	02/07/2020
QuickDBD	*https://www.quickdatabasediagrams.com/*	15/01/2021
ZDOCK	*https://zdock.umassmed.edu/*	01/02/2021
HPEPDOCK	*http://huanglab.phys.hust.edu.cn/hpepdock/*	20/01/2021

Supplementary Table S6. The full list of detected protein, peptide, predicted interaction of proteins, and peptides

Uniprot ID	Gene Name	Score	CI%	#Peptides	Coverage%	MS
Q02156	PRKCE	54	93	13	26	MS
Q9UJ99	CDH22	52	92	7	13	MS
P82979	CIP29/HCC1	52	93	8	55	MS
Q14573	ITPR3	50	87	24	10	MS
P48729	CSNK1A1	49	85	10	28	MS
P56705	WNT4	49	84	6	28	MS
Q13547	HDAC1	47	78	7	21	MS
Q07820	McL1	46	62	7	NA	MS/MS
P62070	RRAS2	44	49	7	37	MS
Q02156	PRKCE	42	22	11	22	MS
P60709	ACTB	41	88	1	28	MS/MS
P14639	**TP53**	**38**	61	21	23	MS/MS
P42574	**Caspase 3**	**36**	87	25	25	MS/MS
PA33942	**PTEN**	**33**	20	8	10	MS/MS
P55210	**Caspase 7**	**29**	28	20	21	MS
Q14790	**Caspase 8**	**29**	48	23	22	MS/MS
P55211	**Caspase 9**	**27**	10	21	26	MS/MS
Q07812	**Bax**	**24**	88	20	21	MS
Q14573	ITPR3	23	88	1	NA	MS/MS
P29597	TYK2	22	88	1	NA	MS/MS
P20749	**BCL2**	**22**	23	7	23	MS/MS
P33151	CDH5	21	84	1	NA	MS/MS
P47736	RAP1GAP	21	89	1	NA	MS/MS
O75084	FZD7	21	82	2	5	MS/MS
Q9UM47	Notch3	20	88	1	NA	MS/MS
P49841	GSK3B	18	61	1	NA	MS/MS
Q9NQ66	PLCB1	18	63	1	NA	MS/MS
Q9NQ66	PLCB1	18	59	1	NA	MS/MS
P48730	CSNK1D (CK1d)	17	64	1	NA	MS/MS
Q9Y6R0	NUMBL	17	62	1	NA	MS/MS
P29597	TYK2	17	83	1	NA	MS/MS
O00755	WNT7A	17	77	1	NA	MS/MS
P09471	GNAO1/GNAO2	17	57	2	10	MS/MS
Q04721	Notch2	16	48	1	NA	MS/MS
O00329	PIK3CD	16	58	1	NA	MS/MS
Q9NQ66	PLCB1	16	40	1	NA	MS/MS

Q15418	RPS6KA1 (RSK1)	16	39	1	NA	MS/MS
Q92569	PIK3R3	15	41	1	NA	MS/MS
P16298	PPP3CB	15	80	1	NA	MS/MS
P61224	RAP1B	15	54	1	NA	MS/MS
P29597	TYK2	15	44	1	NA	MS/MS
Q9BT81	SOX7	15	37	1	NA	MS/MS
P36897	TGFBR1	15	51	1	NA	MS/MS
P63092	GNAS	14	23	1	NA	MS/MS
Q07954	LRP1	14	28	2	1	MS/MS
O75581	LRP6	14	10	1	NA	MS/MS
P10301	RRAS	14	51	1	NA	MS/MS
P29597	TYK2	14	49	1	NA	MS/MS
Q9Y297	BTRCP	14	22	1	NA	MS/MS
P20749	BCL2	12	21	1	NA	MS/MS
Q15349	RPS6KA2 (RSK2)	12	41	1	NA	MS/MS
P35222	CTNNB1	10	17	1	NA	MS/MS
P42338	PIK3CB	9	20	1	NA	MS/MS
O15297	PP2C/WIP1	8	39	1	NA	MS/MS
P35222	CTNNB1	6	11	1	NA	MS/MS
